# Synthesis and Solvatochromism of New Thiazole Derivatives Bearing N,N-Bis(2-methoxyethyl) Benzenesulfonamide Moiety

**DOI:** 10.1007/s10895-025-04430-8

**Published:** 2025-07-08

**Authors:** Dilek Bayramin, Gülsiye Öztürk

**Affiliations:** 1 Department of Chemistry, The Graduate School of Natural and Applied Sciences, Dokuz Eylül University, Tınaztepe Campus, İzmir, 35160 Turkey; 2 Hazelnut Research Institute, Giresun, 28200 Turkey; 3 Department of Chemistry, Faculty of Sciences, Dokuz Eylül University, Tınaztepe Campus, Izmir, 35160 Turkey

**Keywords:** Thiazole, Benzenesulfonamide, Bis(2-methoxyethyl)amine, Hantzsch thiazole synthesis, Solvatochromism

## Abstract

New thiazole derivatives containing *N*,* N*-bis(2-methoxyethyl)benzenesulfonamide groups at the C(4) atom and amino groups bearing electron-donating and electron-withdrawing substituents at the C(2) atom of the heterocycle were obtained via a three-step synthetic method. The sulfonamide intermediate compounds were synthesized with a disubstituted amine of bis(2-methoxyethyl)amine and dibrominated intermediates were obtained via the reaction of bromine. The final step of the Hantzsch thiazole synthesis via different thiourea derivatives yielded the desired thiazole derivatives. In addition, their absorption, emission and solvatochromic properties were examined in several solvents. All compounds presented large Stokes’ shift values within the range of 5020–11,974 cm⁻¹ and excellent photostabilities which are important characteristics of fluorophores. The o-methoxyphenylamino substituted thiazole displayed the largest Stokes’ shift value of 11,974 cm⁻¹ in methanol suggesting that the molecule was highly polarized in the excited state. They are dependent on solvent polarity and generally display bathochromic shifts in polar solvents with a linear correlation in the fluorescence wavenumbers and the Stokes’ shift values versus the empirical parameter values of solvent polarity and solvent polarity parameter values. Notably, the excited state dipole moment was greater than the dipole moment in the ground state. The Kamlet-Taft analysis revealed that the solvatochromic response of thiazole derivatives were closely related to the solvent polarity, ability of H-bonding, and electron-donating and withdrawing groups on the benzene ring. The Catalán model was employed to investigate solvent effects on structural variations among the studied compounds, revealing that the type and position of substituent affected dipolarity, polarizability, and hydrogen bonding capabilities.

## Introduction

Thiazoles or 1,3-thiazoles are a class of 5-membered heterocyclic organic compounds that have both electron-donating nitrogen and sulfur atoms and electron-accepting C = N groups [1]. Thiazoles exhibit greater aromatic character than other azoles due to the effective delocalization of lone pair electrons into the π-electron system [2, 3]. Owing to their significant roles as fluorescent probes, chemosensors, corrosion inhibitors, and biological agents, there has been considerable interest in the development of different thiazole derivatives [1–22] Compounds containing thiazoles have a wide range of biological activities such as anti-inflammatory [4], antibacterial [5], antioxidant [6], analgesic [7], antimicrobial [8], antitumor [9], anticancer [10], anticonvulsant [11], antifungal [12], neuroprotective [13] and antiparasitic [14] activities. Thiazole derivatives have great potential in medical chemistry and play important roles in the discovery of new drug compounds [15–17]. Molecules containing donor-acceptor groups have high quantum yields, large Stokes’ shifts, etc., which are desirable properties for fluorescent probes or sensors [18, 19]. Thiazole derivatives have great potential in the use of fluorescent substances because of their versatile features such as high quantum yields and high Stokes’ shift values [20–22]. The thiazole core is also present in a natural compound named luciferin, which is generated in bioluminescent organisms such as Lampyridae/fireflies [23]. Suntsova et al. synthesized a series of thiazole-based fluorophores with significant photophysical properties such as good quantum yields and large Stokes’ shifts and demonstrated sensitivity to structural and environmental factors [21]. A series of derivatives containing thiazole cores that exhibited fluorescent behavior, such as long emission maxima, good quantum yields and large Stokes’ shifts, were synthesized by Wrona-Piotrowicz et al. [22]. Fluorescent compounds with high quantum yields are suitable for sensitive fluorescence imaging, signaling or sensing [24–27]. Some typical fluorescent molecules such as fluorescein, rhodamine, cyanine, and BODIPY with small Stokes’ shifts can reabsorb the emitted photons [28]. For the purpose of alleviating self-quenching which originates from molecular self-absorption, designing fluorescent compounds with large Stokes’ shift values is important [28–30]. A novel fluorescent probe with a large Stokes’ shift and high fluorescence quantum yield was designed by Gan and coworkers [30]. A fluorescent molecule with good photostability is a photophysical property that is also desirable. Small organic fluorescent molecules have relatively low photostability. Therefore, favorable photostability can be achieved by enhancing the conjugated bands of fluorescent molecules [29, 31] or the addition of electron withdrawing and electron donating substituents to the molecules to change the energy level of the singlet and triplet states [32]. Sulfonamide is an electron-withdrawing functional group. Compounds substituted with sulfonamide groups have been developed as cation and anion chemosensors because they have characteristic such as being hydrogen bond donors and having intermolecular and intramolecular hydrogen bonds [33–35]. Fluorescent probes containing sulfonamide groups, which increase their solubility in water, have been developed for the detection of metal ions [36].

Solvatochromic investigations have revealed possible applications in optical light-emitting diodes, solvent polarity determination, the identification of explosives via colorimetric chemosensors, dye-sensitized solar cells, photoluminescent materials, and volatile organic materials for laser applications [37, 38]. To better understand the excited state, the dependence of the absorption and emission characteristics of organic molecules on solvent polarity can be studied [39–41]. Solvatochromic investigations are carried out to explain the nonlinear optical properties of organic substances, which are primarily expressions of an excited state nature. If hydrogen bonds between solute and solvent molecules exist, the use of continuum models based on the theory of dielectrics will be limited. Currently, for quantitative explanations of solvatochromism, different types of polarity scales such as empirical parameter values of solvent polarity (E_T_(30)) and solvent polarity parameter values (E_T_^N^) are used.

**Fig. 1:**
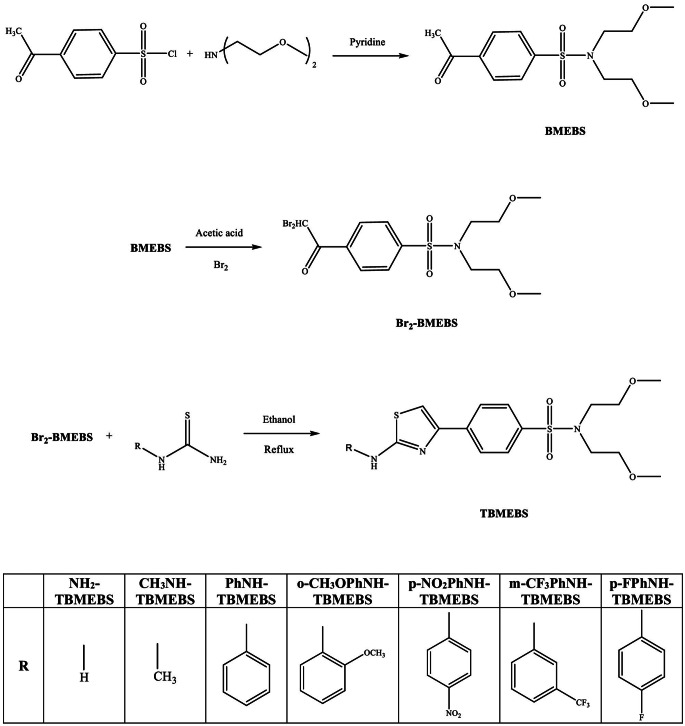
The synthetic pathway of TBMEBS derivatives

In this study, the *N*,* N*-disubstituted-4-acetylbenzenesulfonamide bearing thiazole derivatives (TBMEBS) of 4-(2-aminothiazol-4-yl)-*N*,* N*-bis(2-methoxyethyl)benzenesulfonamide (NH_2_-TBMEBS), *N*,* N*-bis(2-methoxyethyl)-4-(2-(methylamino)thiazol-4-yl)benzenesulfonamide (CH_3_NH-TBMEBS), *N*,* N*-bis(2-methoxyethyl)-4-(2-(phenylamino)thiazol-4-yl)benzenesulfonamide (PhNH-TBMEBS), *N*,* N*-bis(2-methoxyethyl)-4-(2-((2-methoxyphenyl)amino)thiazol-4-yl)benzenesulfonamide (o-CH_3_OPhNH-TBMEBS), *N*,* N*-bis(2-methoxyethyl)-4-(2-((4-nitrophenyl)amino)thiazol-4-yl)benzenesulfonamide (p-NO_2_PhNH-TBMEBS), *N*,* N*-bis(2-methoxyethyl)-4-(2-((3-(trifluoromethyl)phenyl)amino)thiazol-4-yl)benzenesulfonamide (m-CF_3_PhNH-TBMEBS) and 4-(2-((4-fluorophenyl)amino)thiazol-4-yl)-*N*,* N*-bis(2-methoxyethyl)benzenesulfonamide (p-FPhNH-TBMEBS) were obtained with the reaction of bis(2-methoxyethyl)amine and different thiourea derivatives (Fig. 1). The photophysical and solvatochromic properties of the new thiazole derivatives were examined in eight different solvents: n-hexane, toluene, tetrahydrofuran, ethyl acetate, chloroform, acetonitrile, ethanol, and methanol.

## Experimental

### Materials and Methods

4-acetylbenzenesulfonyl chloride, bis(2-methoxyethyl)amine, bromine, sodium bicarbonate, sodium sulfate, thiourea, *N*-methylthiourea, *N*-phenylthiourea, 1-(2-methoxyphenyl)-2-thiourea, 1-(4-nitrophenyl)-2-thiourea, [3-(trifluoromethyl)phenyl]thiourea, (4-fluorophenyl)thiourea, and quinine hemisulfate salt monohydrate were purchased from Sigma-Aldrich. Thin-layer chromatography (TLC) was performed on silica gel 60 F_254_ plates from Merck. Pyridine was supplied from Sigma-Aldrich and purified according to the given method [42]. Ethanol, tert-butyl methyl ether, ethyl acetate, hexane and sulfuric acid were purchased from Sigma-Aldrich and chloroform was purchased from Merck. A molecular sieve (88 − 12 mesh, 3 A) was purchased from Sigma-Aldrich. Toluene, tetrahydrofuran, ethyl acetate, chloroform, acetonitrile, ethanol, and methanol were of spectroscopic grade and were obtained from Merck. Hexane was purchased from Sigma-Aldrich. Column chromatography purifications were carried out with 70–230 mesh silica gel (0.063–0.2 mm, Merck).

The structures of the synthesized compounds were characterized by FT-IR, ^1^H-NMR, ^13^C-NMR, and LC-QTOF-MS. Infrared spectroscopy was carried out on a Perkin Elmer FTIR spectrophotometer (spectra BX-II) as KBr discs. ^1^H-NMR and ^13^C-NMR measurements were recorded on a Varian Mercury AS-400 (400 MHz and 100 MHz, respectively) spectrometer using CDCl_3_ as the solvent. For the LC-QTOF-MS method, chromatographic separation was performed via Agilent 1260 Infinity series HPLC system (Agilent Technologies, Santa Clara, CA, USA) equipped with a dual pump, degasser and auto sampler and MS analysis was performed via an Agilent 6550 iFunnel high-resolution Accurate Mass QTOF-MS system equipped with an Agilent Dual Jet Stream electrospray ionization (Dual AJS ESI) interface. The UV-visible absorption spectra were obtained with a Shimadzu UV-1800 spectrophotometer. The emission spectra were obtained on a Varian-Cary Eclipse fluorescence spectrophotometer. All melting points were determined by an electrothermal digital melting point apparatus (Southend, UK) and were uncorrected. The purity of the compounds synthesized was verified by TLC using a Uv-light and their melting points.

For fluorescence quantum yield (Ф_F_) determinations, the optical density of the samples was adjusted to 0.1. The fluorescence quantum yield (Ф_F_) was calculated according to the following Eq. (1) [43–45]:

1 $$\:{\varPhi\:}_{F}={\varPhi\:}_{ref}.\frac{F}{{F}_{ref}}.\frac{{A}_{ref}}{A}.\frac{{n}^{2}}{{{n}_{ref}}^{2}}$$

where F and F_ref_ are the areas of the fluorescence emission spectrum of the samples and the reference at the excitation wavelength, respectively. A and A_ref_ refer to the absorbance of the samples and reference, respectively. n and n_ref_ are the refractive index of the samples and reference, respectively. Quinine hemisulfate was used as a reference (Φ = 0.54, in 0.5 M H_2_SO_4_) [46].

The solvatochromic properties of TBMEBS derivatives were examined according to the correlations of the absorbance wavenumbers (ῡ_a_, cm^− 1^) (Fig. 4), fluorescence wavenumbers (ῡ_f_, cm^− 1^) (Fig. 5), and Stokes’ shift values (Δῡ) (Fig. 6) with the solvent polarizability function ƒ(ε,n), the empirical parameter values of solvent polarity (E_T_(30)), and the solvent polarity parameter values (E_T_^N^). The correlations between the Stokes’ shift values (Δῡ = ῡ_a_ ‒ ῡ_f_) and solvent polarizability (ƒ(ε,n)) parameters were examined via the following Eq. (2) [47, 48]:

2 $${\bar \upsilon _a} - {\bar \upsilon _f} = m.f\left( {\varepsilon,n} \right) + {\rm{const}}$$

where ῡ_a_ and ῡ_f_ are the wavenumbers of the absorption and emission band shifts declared in cm^− 1^ and

3 $$f\left( {\varepsilon,n} \right) = {{2{{\rm{n}}^2} + 1} \over {{{\rm{n}}^2} + 2}} \times \left( {{{\varepsilon - 1} \over {\varepsilon + 2}} - {{{n^2} - 1} \over {{n^2} + 2}}} \right)$$

n and ε are the refractive index and dielectric constant of the solvent [49], respectively.

The E_T_(30) values were obtained for solvents via Eq. (4) and are known as one of the empirical parameters of solvent polarity.

4 $$\:RPM=\:\frac{{E}_{T}\left(n-hexane\right)-{E}_{T}}{{E}_{T}(n-hexane)}$$

A solution of n-hexane was used as the reference because its relative polarity measure (RPM) value is zero [50].

The E_T_^N^ values are known as solvent polarity parameters and were determined by Reichardt according to Eq. (5).

5 $$\:{E}_{T}^{N}=\:\frac{{E}_{T}\left(solvent\right)-\:{E}_{T}\left(TMS\right)}{{E}_{T}\left(water\right)-\:{E}_{T}\left(TMS\right)}=\frac{{E}_{T}\left(solvent\right)-30.7}{32.7}$$

The E_T_^N^ values are dimensionless and defined by the Eq. (5) based on water as the most polar solvent and tetramethylsilane (TMS) as the least polar solvent. The E_T_^N^ scale goes from 0.000 for TMS, which is the least polar solvent, to 1.000 for water, the most polar solvent [51].

The Kamlet-Taft Eq. (6) which is a linear regression equation was used to determine the solvent-solute interactions [52].

6 $$\bar \upsilon = {\bar \upsilon _0} + a\alpha + b\beta + s\pi *$$

where ῡ is the maximum absorption wavenumbers (cm^− 1^) of solute, ῡ_0_ is the maximum absorption wavenumbers (cm^− 1^) of the reference system, α is the hydrogen-bond donating ability of the solvent (acidity), β is the hydrogen-bond accepting ability of solvent (basicity), π* is the solvent dipolarity (polarizability), and a, b, s are the correlation coefficients of α, β, and π* Kamlet-Taft parameters, respectively [53].

The Catalán’s solvent polarity model provides a more detailed solute-solvent interactions. This model describes solvent effects with four empirical parameters. The Catalán Eq. (7) is given below.

7 $$\bar \upsilon = {\bar \upsilon _o} + a.SA + b.SB + c.SdP + d.SP$$

where ῡ is the observed absorption or emission wavenumber of the solute in cm^− 1^, ῡ_o_ is the reference wavenumber of the solute in a non-interacting solvent in cm^− 1^, SA (solvent acidity), SB (solvent basicity), SdP (solvent dipolarity), SP (solvent polarizability), and a, b, c, d are the empirical coefficients of SA, SB, SdP, and SP solvent parameters, respectively [54].

All solvent parameters of ε, n [55], ƒ(ε,n), E_T_(30), E_T_^N^, Kamlet-Taft (α, β, π*), and Catalán (SA, SB, SdP, SP) used in these studies are summarized in Table 1.

**Table 1:** ε, n, *f* (ε, n), E_T_(30), E_T_^N^, Kamlet-Taft (α, β, π*), and Catalán (SA, SB, sdp, SP) studied solvent parameters

Solvent	ε^a^	*n* ^b^	f (ε,*n*)^c^	E_T_(30)^d^	E_T_^Ne^	α^f^	β^g^	π*^h^	SA^i^	SB^j^	SdP^k^	SP^l^
n-Hexane	1.90	1.372	0.0023	30.9	0.009	0.00	0.00	-0.04	0.000	0.056	0.00	0.616
Toluene	2.38	1.494	0.0291	33.9	0.099	0.00	0.11	0.54	0.000	0.128	0.284	0.782
Tetrahydrofuran	7.60	1.404	0.5490	37.4	0.207	0.00	0.55	0.58	0.000	0.591	0.634	0.714
Ethyl acetate	6.02	1.370	0.4892	38.1	0.228	0.00	0.45	0.55	0.000	0.542	0.603	0.656
Chloroform	4.81	1.444	0.3696	39.1	0.259	0.20	0.10	0.53	0.047	0.071	0.614	0.783
Acetonitrile	37.5	1.342	0.8630	46.0	0.460	0.19	0.40	0.75	0.044	0.286	0.974	0.645
Ethanol	24.5	1.359	0.8127	51.9	0.654	0.86	0.75	0.54	0.400	0.658	0.783	0.633
Methanol	32.7	1.326	0.8545	55.9	0.761	0.98	0.66	0.60	0.605	0.545	0.904	0.608

^a^ε = Dielectric constant (20 °C)

^b^n = Refractive index (25 °C)

^c^*f* (ε,n) = Solvent polarizability function

^d^E_T_(30) = Empirical parameter of solvent polarity

^e^E_T_^N^ = Solvent polarity parameter

^f^α = Hydrogen-band donating ability of solvent (acidity)

^g^β = Hydrogen-band accepting ability of solvent (basicity)

^h^π* = Solvent polarizability

^i^SA = Solvent acidity

^j^SB = Solvent basicity

^k^SdP = Solvent polarizability

^l^SP = Solvent dipolarity

The photostabilities of the TBMEBS derivatives were measured in solutions of eight solvents following 1 h of continuous illumination on a fluorescence spectrophotometer equipped with light from a high power Xe lamp.

### Synthesis of Intermediates

The synthesis of 4-acetyl-*N*,* N*-bis(2-methoxyethyl)benzenesulfonamide (BMEBS) was carried out via a reported procedure (Fig. 1) [56, 57]. Bis(2-methoxyethyl)amine (0.675 mL, 0.457 mmol) was dissolved and stirred in pyridine (10 mL) for almost 30 min at room temperature. The resulting solution was cooled to 0–5 °C. 4-acetylbenzenesulfonyl chloride (1 g, 0.457 mmol) was added in portions. The solution was stirred for 2–3 h at 0 °C. The reaction mixture was stirred at room temperature overnight. Then, ice-embedded samples were added. The resulting reaction mixture was extracted with 3 × 30 mL of chloroform. The resulting residue was chromatographed on silica gel using 1:2 ethyl acetate/n-hexane as the eluent. The compound BMEBS was obtained as a white solid. The yield was 77%. mp: 41–42 °C. FT-IR (KBr, υ_max_, cm^− 1^): 2923 (aliphatic C–H), 2852 (OCH_2_), 1691 (C = O), 1596 (aromatic ring C − C), 1345 (asymmetric SO_2_), 1160 (symmetric SO_2_), 1118 (C–O), 1093 (aliphatic C–N), 941 (S–N). ^1^H NMR (400 MHz, CDCl_3_, δ, ppm): 8.05 ‒ 8.03 (d, *J* = 8.8 Hz, 2 H, Ar‒H), 7.92 ‒ 7.90 (d, *J* = 8.4 Hz, 2 H, Ar‒H), 3.51 ‒ 3.49 (t, *J* = 6.4 Hz, 4 H, -O–CH_2_-), 3.44 − 3.41 (t, *J* = 5.6 Hz, 4 H, -N–CH_2_-), 3.25 (s, 6 H, CH_3_–OR), 2.63 (s, 3 H, CH_3_–CO-).

The synthesis of 4-(2,2-dibromoacetyl)-*N*,* N*-bis(2-methoxyethyl)benzenesulfonamide (Br_2_-BMEBS) was carried out via a previously reported procedure (Fig. 1) [58]. 4-acetyl-*N*,* N*-bis(2-methoxyethyl)benzene sulfonamide (0.360 g, 1.141 mmol) was stirred in acetic acid (~ 10 mL) at room temperature for 30 min. Bromine (0.064 mL, 1.241 mmol) was added at 0–5 °C. The reaction mixture was stirred at room temperature overnight. The mixture was quenched on ice. Then, it was extracted to methyl tert-butyl ether (50 mL). The organic layer was washed with aq. 10% NHCO_3_ solution and then with water (50 mL) and dried over Na_2_SO_4_ and filtered. After the crude product was obtained, it was purified by column chromatography on silica gel using 4:3 ethyl acetate/n-hexane as the eluent. The Br_2_-BMEBS compound was obtained as an off-white solid. (Fig. 1). The yield was 91%. mp: 55–56 °C. FT-IR (KBr, υ_max_, cm^− 1^): 3092 (aromatic C–H), 2922 (aliphatic C–H), 2853 (OCH_2_), 1709 (C = O), 1329 (asymmetric, SO_2_), 1277 (symmetric, SO_2_), 1157 (C–O), 1114 (aliphatic, C–N), 1035 (C–S–C), 855 (S–N), 659 (C–Br). ^1^H NMR (400 MHz, CDCl_3_, δ, ppm): 8.20 ‒ 8.18 (d, *J* = 8.4, 2 H, Ar‒H), 7.95 ‒ 7.93 (d, *J* = 8.4 Hz, 2 H, Ar‒H), 6.65 (s, 1H, Br_2_–CH-), 3.52 ‒ 3.49 (t, *J* = 6.4 Hz, 4 H, -O–CH_2_-), 3.47 ‒ 3.45 (t, *J* = 6.4 Hz, 4 H, -N–CH_2_-), 3.24 (s, 6 H, CH_3_–OR).

### General Synthesis of TBMEBS Derivatives

The synthesis of thiazole derivatives was achieved via a synthetic procedure reported in the literature (Fig. 1) [5, 59]. Intermediate Br_2_-BMEBS (1 eq.) and the thiourea derivative (1 eq.) were dissolved in anhydrous ethanol (20 mL). The reaction mixture was then refluxed overnight. After the completion of the reaction, which was checked by TLC, the mixture was cooled to room temperature. The mixture was poured into ice-cooled water. The pH of the solution was adjusted to 7 with 10% NaHC_3_. The resulting solid was collected via vacuum filtration. It was then dried and yielded a crude product. The product was purified by column chromatography on silica gel. The amounts of reagents and melting points, FTIR, ^1^H NMR, ^13^C NMR, and mass spectrum data of the thiazole derivatives are reported below:

*Synthesis of 4-(2-aminothiazol-4-yl)-N*,* N-bis(2-methoxyethyl)benzenesulfonamide (NH*_*2*_*-TBMEBS)*: Br_2_-BMEBS (0.480 g, 1.014 mmol) and thiourea (0.077 g, 1.014 mmol) were used. The NH_2_-TBMEBS compound was obtained as a pink solid. The yield was 42%. mp: 83 − 84 ºC. FT-IR (KBr, υ_max_, cm^− 1^): 3417, 3343 (N − H), 3198 (aromatic CH), 2926 (aliphatic CH), 1611 (C = N), 1532 (aromatic ring C − C), 1335 (asymmetric SO_2_), 1156 (symmetric SO_2_), 1117 (C–O), 1090 (aliphatic C–N), 1034 (C–S–C), 934 (S–N). ^1^H NMR (400 MHz, CDCl_3_, δ, ppm): 7.88 ‒ 7.86 (d, *J* = 8.8 Hz, 2 H, Ar‒H), 7.81 ‒ 7.79 (d, *J* = 8.8 Hz, 2 H, Ar‒H), 6.84 (s, 1H, S–CH = C), 5.13 (s, 2 H,–NH_2_), 3.52 ‒ 3.49 (t, *J* = 6.0 Hz, 4 H, -O–CH_2_-), 3.40 ‒ 3.37 (t, *J* = 6.0 Hz, 4 H, -N–CH_2_-), 3.27 (s, 6 H, CH_3_–OR). ^13^C NMR (100 MHz, CDCl_3_, δ, ppm): 167.5, 149.5, 138.4, 138.4, 127.5, 126.3, 105.4, 71.5, 58.7, 48.6. LC-QTOF-MS (m/z): Calcd. for C_15_H_21_N_3_O_4_S_2_ [M]^+^, 371.47; found, 372.11 [M + 1]^+^.

*Synthesis of N*,* N-bis(2-methoxyethyl)-4-(2-(methylamino)thiazol-4-yl)benzenesulfonamide (CH*_*3*_*NH-TBMEBS)*: Br_2_-BMEBS (0.441 g, 0.932 mmol) and *N*-methylthiourea (0.084 g, 0.932 mmol) were used. The CH_3_NH-TBMEBS compound was obtained as a pale pink solid. The yield was 61%. mp: 83–84 °C. FT-IR (KBr, υ_max_, cm^− 1^): 3213 (N − H), 3109 (aromatic C–H), 2924 (aliphatic C–H), 2891 (O–CH_2_), 1582 (C = N), 1342 (asymmetric SO_2_), 1153 (symmetric SO_2_), 1122 (C–O), 1088 (aliphatic C–N), 1062 (C–S–C), 962 (S–N). ^1^H NMR (400 MHz, CDCl_3_, δ, ppm): 7.92 ‒ 7.90 (d, *J* = 8.4 Hz, 2 H, Ar‒H), 7.82 ‒ 7.80 (d, *J* = 8.0 Hz, 2 H, Ar‒H), 6.85 (s, 1H, S–CH = C), 5.47 (s, 1H,–NH), 3.53 ‒ 3.51 (t, *J* = 6.0 Hz, 4 H, -O–CH_2_-), 3.41 ‒ 3.38 (t, *J* = 5.6 Hz, 4 H, -N–CH_2_-), 3.29 (s, 6 H, CH_3_–OR), 3.01 (s, 3 H, CH_3_–N-). ^13^C NMR (100 MHz, CDCl_3_, δ, ppm): 170.8, 149.8, 138.7, 138.4, 127.5, 126.3, 103.5, 71.5, 58.7, 48.6, 32.2. LC-QTOF-MS (m/z): Calcd. for C_16_H_23_N_3_O_4_S_2_^+^ [M]^+^ 385.50; found, 386.13 [M + 1]^+^.

*Synthesis of N*,* N-bis(2-methoxyethyl)-4-(2-(phenylamino)thiazol-4-yl)benzenesulfonamide (PhNH-TBMEBS)*: Br_2_-BMEBS (0.270 g, 0.571 mmol) and *N*-phenylthiourea (0.087 g, 0.571 mmol) were used. The PhNH-TBMEBS compound was obtained as a dark brown solid. The yield was 63%. mp: 65–66 °C. FT-IR (KBr, υ_max_, cm^− 1^): 3330 (N − H), 3103 (aromatic C–H), 2921 (aliphatic C–H), 2851 (O–CH_2_), 1597 (C = N), 1547 (aromatic ring C–C), 1329 (asymmetric SO_2_), 1155 (symmetric SO_2_), 1114 (C–O), 1088 (aliphatic C–N), 1057 (C–S–C), 935 (S–N). ^1^H NMR (400 MHz, CDCl_3_, δ, ppm): 8.05 (s, 1H,–NH), 7.95 ‒ 7.93 (d, *J* = 8.4 Hz, 2 H, Ar‒H), 7.84 ‒ 7.82 (d, *J* = 8.4 Hz, 2 H, Ar‒H), 7.42 ‒ 7.40 (dd, *J* = 7.6, 7.2 Hz, 2 H, Ar‒H), 7.38 ‒ 7.36 (d, *J* = 7.2 Hz, 2 H, Ar‒H), 7.11 ‒ 7.07 (t, *J* = 6.8 Hz, 1H, Ar‒H), 6.93 (s, 1H, S–CH = C), 3.54–3.51 (t, *J* = 6.4 Hz, 4 H, -O–CH_2_-), 3.42 ‒ 3.39 (t, *J* = 5.6 Hz, 4 H, -N–CH_2_-), 3.28 (s, 6 H, CH_3_–OR). ^13^C NMR (100 MHz, CDCl_3_, δ, ppm): 165.1, 149.0, 139.9, 138.7, 138.0, 129.5, 127.6, 126.4, 123.5, 118.5, 104.2, 71.5, 58.8, 48.6. LC-QTOF-MS (m/z): Calcd. for C_21_H_25_N_3_O_4_S_2_^+^ [M]^+^, 447.57; found, 448.14 [M + 1]^+^.

*Synthesis of N*,* N-bis(2-methoxyethyl)-4-(2-((2-methoxyphenyl)amino)thiazol-4-yl)benzenesulfonamide (o-CH*_*3*_*OPhNH-TBMEBS)*: Br_2_-BMEBS (0.640 g, 1.353 mmol) and 1-(2-methoxyphenyl)-2-thiourea (0.246 g, 1.353 mmol) were used. The o-CH_3_OPhNH-TBMEBS compound was obtained as a pale-yellow solid. The yield was 45%. mp: 106–107 °C. FT-IR (KBr, υ_max_, cm^− 1^): 3343 (N − H), 3109 (aromatic C − H), 2924 (aliphatic C − H), 2851 (O–CH_2_), 1602 (C = N), 1546 (aromatic ring C–C), 1337 (asymmetric SO_2_), 1157 (symmetric SO_2_), 1113 (C–O), 1088 (aliphatic C–N), 1060 (C–S–C), 937 (S–N). ^1^H NMR (400 MHz, CDCl_3_, δ, ppm): 8.13 ‒ 8.12 (d, *J* = 7.2 Hz, 1H, Ar‒H), 8.01 ‒ 7.99 (d, *J* = 8.8 Hz, 2 H, Ar‒H), 7.87 ‒ 7.85 (d, *J* = 8.4 Hz, 2 H, Ar‒H), 7.81 (s, 1H,–NH), 7.05 ‒ 7.04 (dd, *J* = 4.8, 4.8 Hz, 1H, Ar‒H), 7.04 ‒ 7.02 (dd, *J* = 4.8, 5.2 Hz, 1H, Ar‒H), 6.98 (s, 1H, S–CH = C), 6.94 ‒ 6.92 (d, *J* = 7.6 Hz, 1H, Ar‒H), 3.93 (s, 3 H, CH_3_OAr), 3.55 ‒ 3.52 (t, *J* = 6.0 Hz, 4 H, -O–CH_2_-), 3.43–3.41 (t, *J* = 6.0 Hz, 4 H, -N–CH_2_-), 3.30 (s, 6 H, CH_3_–OR). ^13^C NMR (100 MHz, CDCl_3_, δ, ppm): 163.9, 149.5, 147.5, 138.6, 138.3, 129.7, 127.6, 126.4, 122.4, 121.1, 116.6, 110.2, 104.4, 71.5, 58.7, 55.8, 48.6. LC-QTOF-MS (m/z): Calcd. for C_22_H_27_N_3_O_5_S_2_^+^ [M]^+^ 477.59; found, 478.15 [M + 1]^+^.

*Synthesis of N*,* N-bis(2-methoxyethyl)-4-(2-((4-nitrophenyl)amino)thiazol-4-yl)benzenesulfonamide (p-NO*_*2*_*PhNH-TBMEBS)*: Br_2_-BMEBS (0.275 g, 0.581 mmol) and 1-(4-nitrophenyl)-2-thiourea (0.115 g, 0.581 mmol) were used. The p-NO_2_PhNH-TBMEBS compound was obtained as a bright yellow solid. The yield was 63%. mp: 213–214 °C. FT-IR (KBr, υ_max_, cm^− 1^): 3312 (N − H), 3106 (aromatic C–H), 2921 (aliphatic C–H), 1597 (C = N), 1531 (asymmetric NO), 1325 (symmetric NO), 1300 (asymmetric SO_2_), 1152 (symmetric SO_2_), 1111 (C–O), 1088 (aliphatic C–N), 1071 (C–S–C), 931 (S–N). ^1^H NMR (400 MHz, CDCl_3_, δ, ppm): 8.26 ‒ 8.24 (d, *J* = 9.2 Hz, 2 H, Ar‒H), 7.97 ‒ 7.95 (d, *J* = 8.4 Hz, 2 H, Ar‒H), 7.90 (s, 1H,–NH), 7.87 ‒ 7.85 (d, *J* = 8.4 Hz, 2 H, Ar‒H), 7.68 ‒ 7.66 (d, *J* = 9.2 Hz, 2 H, Ar‒H), 7.11 (s, 1H, S–CH = C), 3.55 ‒ 3.52 (t, *J* = 6.0 Hz, 4 H, -O–CH_2_-), 3.43 ‒ 3.40 (t, *J* = 5.6 Hz, 4 H, -N–CH_2_-), 3.28 (s, 6 H, CH_3_–OR). ^13^C NMR (100 MHz, CDCl_3_, δ, ppm): 161.7, 150.0, 145.6, 141.9, 138.9, 137.9, 127.7, 126.4, 125.7, 116.3, 106.4, 71.4, 58.8, 48.6. LC-QTOF-MS (m/z): Calcd. for C_21_H_24_N_4_O_6_S_2_^+^ [M]^+^ 492.57, found: 493.13 [M + 1]^+^.

*Synthesis of N*,* N-bis(2-methoxyethyl)-4-(2-((3-(trifluoromethyl)phenyl)amino)thiazol-4-yl)benzenesulfonamide (m-CF*_*3*_*PhNH-TBMEBS)*: Br_2_-BMEBS (0.250 g, 0.528 mmol) and [3-(trifluoromethyl)phenyl]thiourea (0.116 g, 0.528 mmol) were used. The m-CF_3_PhNH-TBMEBS compound was obtained as a pale-yellow solid. The yield was 66%. mp: 90 − 91 ºC. FT-IR (KBr, υ_max_, cm^− 1^): 3266 (N − H), 3101 (aromatic CH), 2934 (aliphatic CH), 2876 (O–CH_2_), 1609 (C = N), 1541 (aromatic ring C–C), 1340 (asymmetric SO_2_,), 1161 (symmetric SO_2_), 1118 (C–O), 1068 (aliphatic C–N), 1039 (C–S–C), 939 (S–N). ^1^H NMR (400 MHz, CDCl_3_, δ, ppm): 8.14 (s, 1H,–NH), 7.94 ‒ 7.92 (d, *J* = 8.8 Hz, 2 H, Ar‒H), 7.93 (s, 1H, Ar–H), 7.84 ‒ 7.82 (d, *J* = 8.8 Hz, 2 H, Ar‒H), 7.64 ‒ 7.62 (d, *J* = 8.4 Hz, 1H, Ar‒H), 7.44 ‒ 7.40 (dd, *J* = 8.0, 8.0 Hz, 1H, Ar‒H), 7.27 ‒ 7.25 (d, *J* = 7.2 Hz, 1H, Ar‒H), 6.98 (s, 1H, S–CH = C), 3.54 ‒ 3.51 (t, *J* = 5.6 Hz, 4 H, -O–CH_2_-), 3.43 ‒ 3.40 (t, *J* = 6.0 Hz, 4 H, -N–CH_2_-), 3.27 (s, 6 H, CH_3_–OR). ^13^C NMR (100 MHz, CDCl_3_, δ, ppm): 163.4, 149.5, 140.8, 138.4, 138.3, 129.8, 127.6, 126.3, 120.5, 118.9, 118.9, 114.3, 114.3, 105.2, 71.4, 58.7, 48.6. LC-QTOF-MS (m/z): Calcd. for C_22_H_24_F_3_N_3_O_4_S_2_^+^ [M]^+^, 515.57; found, 516.13 [M + 1]^+^.

*Synthesis of 4-(2-((4-fluorophenyl)amino)thiazol-4-yl)-N*,* N-bis(2-methoxyethyl)benzenesulfonamide (p-FPhNH-TBMEBS)*: Br_2_-BMEBS (0.590 g, 1.247 mmol) and (4-fluorophenyl)thiourea (0.212 g, 1.247 mmol) were used. The p-FPhNH-TBMEBS compound was obtained as a pale brown solid. The yield was 34%. mp: 76 − 77 ºC. FT-IR (KBr, υ_max_, cm^− 1^): 3308 (N − H), 3081 (aromatic C − H), 2923 (aliphatic C − H), 1597 (C = N), 1549 (aromatic ring C − C), 1334 (asymmetric SO_2_), 1152 (symmetric SO_2_), 1116 (C–O), 1088 (aliphatic C–N), 1028 (C–S–C), 931 (S–N). ^1^H NMR (400 MHz, CDCl_3_, δ, ppm): 8.62 (s, 1H, −NH), 7.96 ‒ 7.94 (d, *J* = 8.4 Hz, 2 H, Ar‒H), 7.86 ‒ 7.84 (d, *J* = 8.4 Hz, 2 H, Ar‒H), 7.44 ‒ 7.43 (d, *J* = 4.4 Hz, 2 H, Ar‒H), 7.42 ‒ 7.41 (d, *J* = 4.4 Hz, 2 H, Ar‒H), 6.95 (s, 1H, S–CH = C), 3.55 ‒ 3.52 (t, *J* = 6.4 Hz, 4 H, -O–CH_2_-), 3.43 ‒ 3.40 (t, *J* = 6.0 Hz, 4 H, -N–CH_2_-), 3.30 (s, 6 H, CH_3_–OR). ^13^C NMR (100 MHz, CDCl_3_, δ, ppm): 165.9, 160.3, 149.4, 138.7, 138.1, 136.2, 127.6, 126.4, 120.9, 120.8, 116.3, 116.1, 107.6, 71.5, 58.7, 48.6. LC-QTOF-MS (m/z): Calcd. for C_21_H_24_FN_3_O_4_S_2_^+^ [M]^+^ 465.56; found, 466.13 [M + 1]^+^.

## Results and Discussion

### Synthesis and Characterization of the TBMEBS Derivatives

2,4-disubstituted thiazole derivatives were synthesized and characterized structurally. The presence of acidic protons attached to the C-2 and C-4 positions in the thiazole ring facilitates the reactions at these positions. Thus, the reaction rate formation of 2,4-disubstituted thiazole derivatives with different donor and acceptor groups might have been influenced by the substituents in the substrates with the electronegative carbon atom at position 4 and the electropositive carbon at position 2 within the thiazole ring [60]. NH_2_-TBMEBS, CH_3_NH-TBMEBS, PhNH-TBMEBS, o-CH_3_OPhNH-TBMEBS, p-NO_2_PhNH-TBMEBS, m-CF_3_PhNH-TBMEBS, and p-FPhNH-TBMEBS derivatives were obtained by attaching amino, methylamino, phenylamino, (2-methoxyphenyl)amino, 4-nitrophenylamino, [3-trifluoromethyl]phenyl)amino, and 4-fluorophenylamino groups at position 2 of the thiazole ring, respectively.

The structures of intermediates BMEBS and Br_2_-BMEBS (α,α-dibromoketone) were characterized by FT-IR and ^1^H-NMR analysis. Structural characterizations of TBMEBS derivatives were confirmed by FT-IR, ^1^H-NMR, ^13^C-NMR, and mass spectral techniques. According to the reported literatures, α-monobrominated ketones were commonly used as starting compounds in the Hantzsch thiazole synthesis thus we aimed to synthesize Br-BMEBS.

(α-monobromoketone) as a starting material [5, 59]. However, Br_2_-BMEBS was obtained due to the difficulty of controlling the formation of Br-BMEBS. It is challenging to obtain monobrominated compounds because the reaction usually produces not only monobrominated compounds but also dibrominated ones under laboratory conditions [61]. In the literature, it is stated that α,α-dibromoketones behave as synthetic equivalents of their corresponding α-bromoketones and in fact α,α-dibromoketones are better reagents than α-bromoketones in Hantzsch thiazole synthesis [62]. α,α-dibromoketones have several advantages over α-bromoketones such as being easy to prepare at room temperature and forming only a single product. Therefore, we synthesized Br_2_-BMEBS as starting materials. The 6.65 ppm chemical shift as a singlet indicated the proton of Br_2_–CH. Typically, Br–CH_2_ protons appear at approximately 4.4 ppm [63, 64]. This finding confirmed that α,α-dibromination was obtained instead of α-monobromination.

The synthetic pathway to obtain TBMEBS derivatives is shown in Fig. 1. These thiazole derivatives were synthesized via Hantzsch reaction between *N*,* N*-disubstituted-4-acetylbenzenesulfonamide and *N*-substituted thiourea. The structures of the synthesized thiazole compounds were confirmed by FT-IR, ^1^H NMR, ^13^C NMR, and mass spectrometry, which indicated that the desired compounds were formed. The infrared spectra revealed that CH_3_NH-TBMEBS, PhNH-TBMEBS, o-CH_3_OPhNH-TBMEBS, p-NO_2_PhNH-TBMEBS, m-CF_3_PhNH-TBMEBS, and p-FPhNH-TBMEBS displayed secondary ‒NH stretching vibration frequencies at 3213, 3330, 3343, 3312, 3266, and 3308 cm^− 1^, respectively. The carbonyl peak at 1709 cm^− 1^ of the intermediate Br_2_-BMEBS did not appear in the FT-IR spectrum. The imine group, C = N, stretching bands of the thiazole ring were observed between 1611 and 1582 cm^− 1^ for the TBMEBS derivatives. The C–S–C stretching bands of the thiazole ring appeared at a range of 1071–1028 cm^− 1^ for the TBMEBS compounds.

The ^1^H NMR spectral data are given in the experimental section. The protons of secondary–NHAr usually appear in the 3–6 ppm range [65]. For the TBMEBS derivatives, the proton signals belonging to the secondary–NHAr group were in the range of 5.47 − 8.62 ppm as singlets. NH_2_-TBMEBS had the two proton signals belonging to the primary–NH_2_ group at 5.13 ppm as singlet indicating the formation of amine protons. The electron-withdrawing groups on the benzene ring caused a decrease in the electron density at the nucleus and shifted the chemical shift values of–NH downfield. The electron-donating groups on the benzene ring caused an increase in the electron density and shifted the chemical shift values of the–NH groups to the upfield. The proton signal of S–CH = C in the thiazole ring appeared in the narrow range of 6.84 − 7.11 ppm as a singlet for all the thiazole derivatives. The ^13^C NMR spectral data are given in the experimental section. Aliphatic carbon atoms appeared in the range of 32.21 − 71.52 ppm and aromatic carbon atoms were in the range of 103.47 − 170.75 ppm. The mass spectral data are given in the experimental section. The M + 1 (m/z) peak of the TBMEBS compounds present in the mass spectra matched the M^+^ peak of the desired structures.

### Absorption, Emission, Solvatochromic Behavior and Photostability of the TBMEBS Derivatives

The absorption and emission spectra of the TBMEBS derivatives were investigated in the solvents of n-hexane, toluene, tetrahydrofuran, ethyl acetate, chloroform, acetonitrile, ethanol and methanol (Tables 2 and 3). The maximum absorption wavelengths (λ_abs_^max^), molar absorption coefficients (ε), maximum emission wavelengths (λ_emis_^max^), Stokes’ shifts (Δῡ), singlet energies (E_s_), fluorescence quantum yields (Ф_F_), and photostabilities of the TBMEBS compounds were determined at a concentration of 1.0 × 10^− 5^ M (Tables 2 and 3). The quantum yields (Ф_F_) of the derivatives were determined by using quinine hemisulfate (Φ_F_ = 0.54) salt monohydrate in 0.5 M sulfuric acid (H_2_SO_4_) as a reference.

**Table 2:** Photophysical properties of **NH**_**2**_**-TBMEBS** and **CH**_**3**_**NH-TBMEBS** in solvents with different polarities

Compound	Solvent	λ_abs_^a^ (nm)	ε^b^ (L.mol^− 1^.cm^− 1^)	λ_emis_^c^ (nm)	Δῡ^d^ (cm^− 1^)	E_S_^e^ (kcal.mol^− 1^)	Ф_F_^f^
NH_2_-TBMEBS	n-Hexane	302	13,200	363	5564	72.8	0.0102
	Toluene	303	13,400	397	7814	72.8	0.0100
	Tetrahydrofuran	310	14,000	409	7808	69.4	0.3436
	Ethyl acetate	314	16,800	406	7216	70.2	0.3781
	Chloroform	315	14,300	407	7176	69.7	0.4042
	Acetonitrile	317	18,000	425	8016	67.2	0.4152
	Ethanol	321	17,500	437	8269	65.4	0.4544
	Methanol	318	17,700	441	8770	64.4	0.4050
CH_3_NH-TBMEBS	n-Hexane	317	23,800	377	5020	72.8	0.0050
	Toluene	317	23,400	402	6670	73.0	0.0072
	Tetrahydrofuran	325	19,800	418	6846	68.4	0.2121
	Ethyl acetate	325	19,400	413	6556	69.1	0.1954
	Chloroform	320	18,200	420	7440	69.6	0.1432
	Acetonitrile	325	16,000	439	7990	65.0	0.4704
	Ethanol	325	17,400	449	8498	64.1	0.3906
	Methanol	324	16,600	454	8838	63.0	0.3813

^a^ λ_abs_ = Maximum absorption wavelength (nm)

^b^ε = Molar absorption coefficient (L.mol^− 1^.cm^− 1^) at λ_max_^abs^

^c^λ_emis_ = Maximum emission wavelength (nm)

^d^Δῡ = Stokes shift (cm^− 1^)

^e^Es = Singlet energy (kcal.mol^− 1^)

^f^Ф_F_ = Fluorescence quantum yield

**Table 3:** Photophysical properties of PhNH-TBMEBS, o-CH_3_OPhNH-TBMEBS, p-NO_2_PhNH-TBMEBS, m-CF_3_PhNH-TBMEBS, and p-FPhNH-TBMEBS in solvents with different polarities

Compound	Solvent	λ_abs1_^a^ (nm)	λ_abs2_^b^ (nm)	ε_1_^c^ (L.mol^− 1^.cm^− 1^)	ε_2_^d^ (L.mol^− 1^.cm^− 1^)	λ_emis_^e^ (nm)	Δῡ^f^ (cm^− 1^)	E_S_^g^ (kcal.mol^− 1^)	Ф_F_^h^
PhNH-TBMEBS	n-Hexane	303	325	27,000	11,000	359	5148	69.1	0.0054
	Toluene	304	326	24,000	11,300	396	7642	72.1	0.0053
	Tetrahydrofuran	303	329	20,300	9200	413	8790	71.7	0.0769
	Ethyl acetate	302	326	19,000	9700	412	8841	69.4	0.0462
	Chloroform	304	327	22,700	9300	415	8798	67.9	0.0306
	Acetonitrile	305	327	23,600	9600	441	10,111	64.7	0.2064
	Ethanol	304	330	25,600	10,200	443	10,321	64.4	0.2040
	Methanol	304	330	22,000	10,700	456	10,964	62.6	0.2523
o-CH_3_OPhNH-TBMEBS	n-Hexane	292	326	38,500	1600	356	6156	72.2	0.0099
	Toluene	306	327	37,000	1500	403	7866	72.0	0.0069
	Tetrahydrofuran	306	328	27,200	1100	428	9315	66.8	0.0039
	Ethyl acetate	292	327	40,700	1600	430	10,990	67.6	0.0093
	Chloroform	302	327	36,800	1300	430	9856	67.6	0.0199
	Acetonitrile	305	328	10,300	1800	470	11,510	61.0	0.0151
	Ethanol	303	331	29,800	1400	473	11,861	61.1	0.0324
	Methanol	304	334	29,900	1600	478	11,974	59.7	0.0318
p-NO_2_PhNH-TBMEBS	n-Hexane	321	357	18,600	22,000	401	6214	56.1	0.0027
	Toluene	322	369	14,300	17,300	474	9958	58.8	0.0016
	Tetrahydrofuran	323	375	13,400	20,300	496	10,798	57.2	0.0029
	Ethyl acetate	325	370	13,000	19,400	493	10,485	57.9	0.0016
	Chloroform	327	376	11,900	18,300	505	10,779	55.6	0.0014
	Acetonitrile	323	374	12,000	18,800	501	10,999	52.6	0.0015
	Ethanol	327	375	12,900	18,000	465	9075	62.2	0.0015
	Methanol	328	375	13,900	20,700	491	10,121	59.1	0.0014
m-CF_3_PhNH-TBMEBS	n-Hexane	281	301	19,400	19,200	354	7338	72.0	0.0069
	Toluene	282	302	19,500	46,000	383	9351	72.8	0.0052
	Tetrahydrofuran	288	302	59,600	50,800	397	9533	74.9	0.0182
	Ethyl acetate	287	304	41,200	42,400	395	9526	72.2	0.0107
	Chloroform	281	303	53,200	44,600	398	10,461	71.2	0.0101
	Acetonitrile	287	306	37,200	41,600	418	10,919	68.1	0.0541
	Ethanol	285	303	62,400	49,600	419	11,221	68.1	0.0850
	Methanol	286	304	49,000	45,800	426	11,490	66.8	0.0912
p-FPhNH-TBMEBS	n-Hexane	306	317	14,500	14,000	374	5941	78.8	0.0064
	Toluene	305	327	16,800	16,400	394	7406	74.1	0.0089
	Tetrahydrofuran	306	324	16,900	7800	414	8525	68.4	0.0603
	Ethyl acetate	307	327	10,200	13,700	411	8242	69.6	0.0642
	Chloroform	308	326	14,100	15,400	418	8544	68.6	0.0210
	Acetonitrile	308	327	14,100	16,900	445	9995	64.1	0.2805
	Ethanol	309	330	13,900	16,900	446	9940	64.5	0.3033
	Methanol	307	333	14,700	16,800	455	10,595	62.6	0.3050

^a^λ_abs1_ = First maximum absorption wavelengths (nm)

^b^λ_abs2_ = Second maximum absorption wavelengths (nm)

^c^ε_1_ = Molar absorption coefficient (L.mol^− 1^.cm^− 1^) at λ_max_^abs1^

^d^ε_1_ = Molar absorption coefficient (L.mol^− 1^.cm^− 1^) at λ_max_^abs2^

^e^λ_emis_ = Maximum emission wavelength (nm)

^f^Δῡ = Stokes shift (cm^− 1^)

^g^Es = Singlet energy (kcal.mol^− 1^)

^h^Ф_F_ = Fluorescence quantum yield

**Fig. 2:**
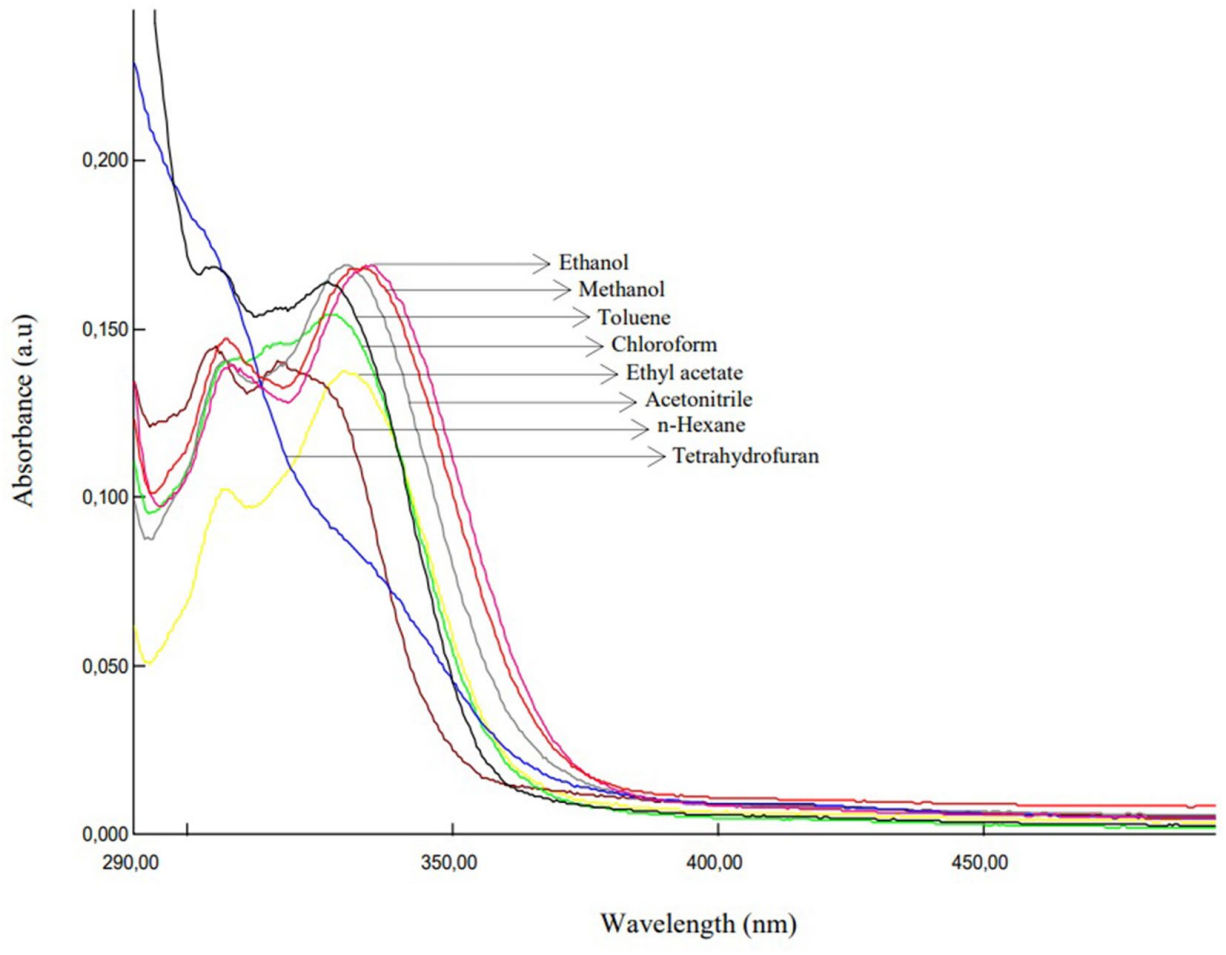
The absorption spectra of p-FPhNH-TBMEBS in different polarity solvents with the concentration of 1.0 × 10− 5 M

**Fig. 3:**
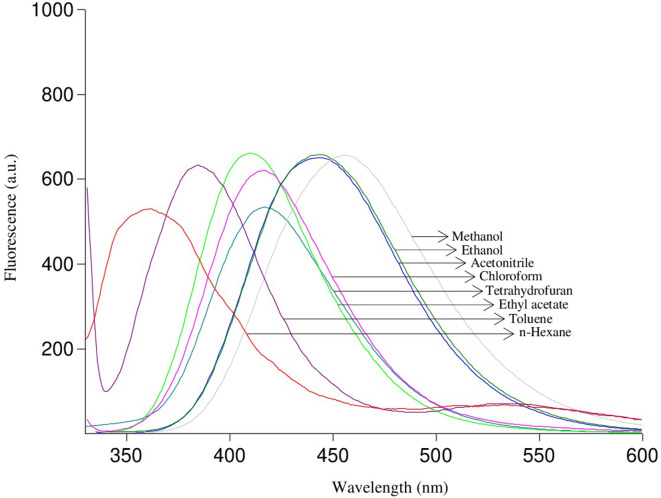
The emission spectra of p-FPhNH-TBMEBS in different polarity solvents with the concentration of 1.0 × 10-5 M

The benzenesulfonamide moiety is a weak meta-directing group. However, when the benzenesulfonamide group was bonded to the thiazole ring, notable changes in fluorescence were observed due to extended π-conjugation. The thiazole ring contains heteroatoms such as doubly bonded nitrogen,–N=, and singly bonded sulfur,–S–. The–N = deactivates the ring by drawing π-electrons towards it and the effect of–N = is greater than that of–S–. Thus, the heterocyclic system becomes nonfluorescent. If the effect on the π-electron system of substituents is greater than on–N=, thiazoles with these substituents may be fluorescent [66]. Therefore, we synthesized thiazole derivatives bearing substituents at one end of the molecule stronger than–N=. Furthermore, among other azole compounds, thiazole has attracted increasing attention because of its planar ring structure and π-electron delocalization of the lone pair of sulfur atoms, which maintains the 6π-electron system and contributes to the aromaticity of the molecule. It is stated that thiazole and its derivatives have exhibited more aromatic structure than five-membered azole heterocyclic compounds did [67]. The photophysical properties of the synthesized thiazole compounds are given in Tables 2 and 3. The absorption and emission spectra of p-FPhNH-TBMEBS are given in Figs. 2 and 3, respectively, as examples. NH_2_-TBMEBS and CH_3_NH-TBMEBS which incorporate–H and–CH_3_, respectively as–R (Ar) groups, presented one absorption maximum between 302 and 325 nm, whereas the remaining derivatives, which have different aryl groups, such as the–R (Ar) substituted group, presented two absorption maxima: the first between 281 and 325 nm and the second between 301 and 376 nm (Tables 2 and 3). When electron-donating substituents such as --NH_2_ and -CH_3_–NH were placed directly at the electropositive carbon at position 2 between heteroatoms in the thiazole ring for NH_2_-TBMEBS and CH_3_NH-TBMEBS, respectively between heteroatoms, it was concluded that the photophysical properties were enhanced by increasing the basicity of the molecules. (Table 2) [67]. The first and second absorption maxima are considered to belong to the π–π^*^ transition of the phenyl group conjugation at the end of the molecule and the n–π^*^ transition of the imine group (–C = N–) conjugation in the thiazole core, respectively [68]. The π–π* transitions occur at smaller wavelengths requiring more energy, in which an electron is excited from the π bonding orbital (HOMO) to the π* antibonding orbital (LUMO). The n–π* transitions occur at higher wavelengths requiring less energy, in which an electron is excited from a non-bonding orbital (HOMO) containing a lone pair of electrons to the π* antibonding orbital (LUMO) [69]. The dipole moments occur when there is a separation of charge and is a measure of the polarity of the molecule. Presumably, the excited state dipole moment is greater than the dipole moment in the ground state. The second absorption maxima may result from charge transfer transitions, where electrons move between different regions of a molecule containing heteroatoms such as nitrogen or oxygen. The synthesized thiazole derivatives presented one emission maximum in the range of 354–505 nm. The red-shifted absorption and emission maxima were observed for p-NO_2_PhNH-TBMEBS at 376 nm and 505 nm, respectively (Table 3), presumably due to the electron-withdrawing and conjugative group of nitro- at benzene ring, which enhanced delocalization and hence increased the length of the conjugated bridge. Among all thiazole derivatives the m-CF_3_PhNH-TBMEBS derivative exhibited the smallest absorption and emission maxima. This molecule contains the trifluoromethyl group (-CF_3_) at one end which is a strong electron-withdrawing and non π-conjugative group, and a sulfonamide group at the other end which is also an electron-withdrawing group. The presence of these two groups might lead to a cross-conjugation which might shorten the conjugation pathway resulting in blue-shifted absorption and emission maxima of this molecule than the other thiazole compounds [70]. Although both nitro and trifluoromethyl groups behave as an electron-withdrawing group, the position of -NO_2_ and -CF_3_ at benzene as para and meta-substituent affect the photophysical properties. The p-NO_2_ group participates in resonance enhancing the conjugated system and causes a lower HOMO-LUMO energy gap and red-shifted absorption and emission. Conversely, the m-CF_3_ group influences the conjugated system only inductively leading to a high HOMO-LUMO energy gap and blue-shifted absorption and emission [71]. The compounds NH_2_-TBMEBS, CH_3_NH-TBMEBS, PhNH-TBMEBS, and p-FPhNH-TBMEBS apart from p-NO_2_PhNH-TBMEBS, m-CF_3_PhNH-TBMEBS, and o-CH_3_OPhNH-TBMEBS presented promising fluorescence quantum yield values (Ф_F_) (Tables 2 and 3). The compounds NH_2_-TBMEBS, CH_3_NH-TBMEBS, PhNH-TBMEBS, and p-FPhNH-TBMEBS displayed quantum yields in the range of 0.20–0.47 in the solvents such as acetonitrile, ethanol, and methanol. On the contrary, p-NO_2_PhNH-TBMEBS, m-CF_3_PhNH-TBMEBS, and o-CH_3_OPhNH-TBMEBS showed lower fluorescence quantum yield values (Ф_F_) in the range of 0.0014–0.0912. The electron-withdrawing groups of p-NO_2_PhNH-TBMEBS and m-CF_3_PhNH-TBMEBS could affect the excitation of the singlet and triplet excited states in organic molecules resulting in lower quantum yield values [72]. p-NO_2_PhNH-TBMEBS presented the lowest fluorescence quantum yield values (Ф_F_), presumably because of rapid intersystem crossing, which probably quenched the singlet excited state and caused low quantum yields of this molecule [44]. In addition, since the NO_2_ group on the benzene ring is a strong meta-directing group, it withdraws π electrons from the ring and thus has a detrimental effect on the fluorescence quantum yield [66]. o-CH_3_OPhNH-TBMEBS contains the methoxy group in the ortho position which increases internal rotations disrupting molecular planarity and can affect fluorescence negatively [73, 74]. In general, the quantum yield values of the derivatives were greater in polar solvents which can presumably be attributed to excited-state complex formation [75, 76].

NH_2_-TBMEBS and CH_3_NH-TBMEBS displayed high molar extinction coefficient values (ε) in the range of 13,200–23,800 L.mol.cm^− 1^. Other TBMEBS derivatives exhibited two molar extinction coefficient values (ε_1_, ε_2_) at two absorption maxima (λ_max1_^abs^, λ_max2_^abs^), which were also significantly high in the range of 1100–59,600 L.mol.cm^− 1^. In general, it was observed that molecules with limited conjugation exhibited higher molar extinction coefficients at smaller absorption maximum due to the highly energy π–π^*^ transition decreasing the HOMO-LUMO energy gap. Molecules, especially p-NO_2_PhNH-TBMEBS, m-CF_3_PhNH-TBMEBS, and p-FPhNH-TBMEBS with increased conjugation shifted the absorption maximum higher and increased the molar absorption coefficient due to the increased HOMO-LUMO energy gap [69].

The Stokes’ shift values (Δῡ = ῡ_a_– ῡ_f_) (cm^− 1^) of all the compounds, which refer to the difference in the wavenumbers of the absorption maximum (ῡ_a_) and fluorescence emission maximum (ῡ_f_) [28], were studied in eight different solvents with different polarities (Tables 2 and 3). The synthesized derivatives exhibited moderate Stokes’ shift values between 5020 and 11,974 cm⁻¹, suggesting that the molecules in the fluorescent state were highly polarized. The largest Stokes’ shift value of 11,974 cm⁻¹ was determined for the o-methoxyphenyl-substituted thiazole derivative of o-CH_3_OPhNH-TBMEBS in methanol. This observation is in agreement with the literature. It has been reported that the small Stokes’ shift of BODIPY dyes can be increased through the introduction of methoxy groups to phenyl ring. The methoxy group can enhance the electron-donating ability of phenyl ring [77]. When the molecule is excited the methoxyphenylamin group and thiazole ring enable intramolecular charge transfer which causes the large Stokes’ shift [78]. Moreover, the synthesized derivatives could form a hydrogen bond with methanol and hence polarize further at excited state and increase the length of the conjugated bridge. Generally, the Stokes’ shifts became more pronounced as the solvent polarity increased because of the large difference in the dipole moment between the ground state and the excited state. Greater Stokes’ shift values means that the interaction between the solvent and solute in the excited state diverge from that in the ground state [79]. The large Stokes’ shifts could be partly caused by a considerable change in the dipole moment of the molecule when it is excited, along with a loss of energy due to the surrounding solvent molecules [80, 81]. The smaller Stokes’ shift values presumably occurred when the molecules tended to quench faster because of energy transfer [82, 83]. The large Stokes’ shift values in the range of 8269–11,974 cm^− 1^ in polar protic solvents such as ethanol and methanol may be due to the solute-solvent interactions, which could be attributed to the formation of hydrogen bonds with the hydroxyl groups of alcohols. Moreover, in general, larger Stokes’ shift values were observed in more polar solvents than in lower-polarity solvents, which could be a result of intermolecular charge transfer as well as dipole-dipole interactions [48].

Furthermore, the solvatochromic and solvatofluoric behaviors of the TBMEBS derivatives were investigated through evaluation of their absorption and emission wavelengths and Stokes’ shift values.

**Fig. 4:**
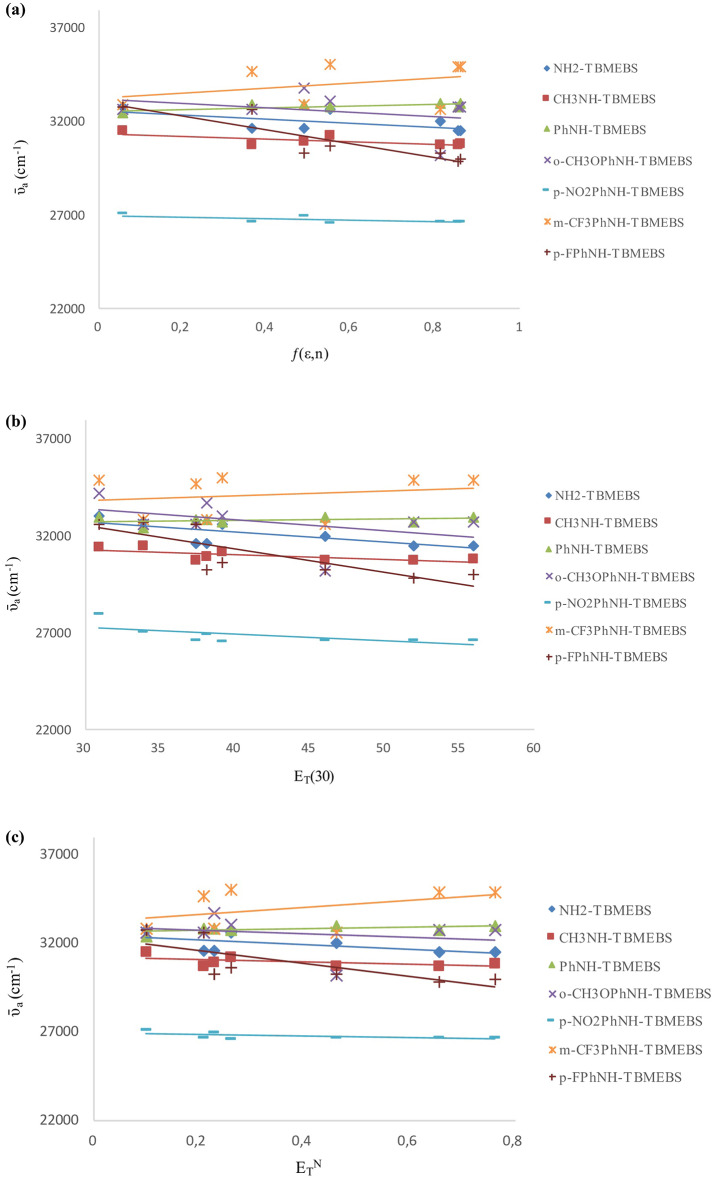
Plots of the absorbance wavenumbers (ῡa, cm− 1) against (**a**) solvent polarizability function ƒ(ε,n) (**b**) empirical parameter values of solvent polarity ET(30) (**c**) solvent polarity parameter values ETN for TBMEBS derivatives in different solvents

The plots of ῡ_a_ (cm^− 1^) against ƒ(ε,n), E_T_(30), and E_T_^N^ were linearly correlated (Fig. 4; Table 4). Three of the parameters studied yielded similar results, i.e., in general, the solvent polarity had a small effect on the absorption wavelengths of the derivatives. As the solvent polarity increased, a slight bathochromic shift in the absorption maxima of the synthesized derivatives was observed. The positive solvatochromism was more pronounced for NH_2_-TBMEBS presumably due to greater polarization of this compound as a result of hydrogen bonding between the–NH_2_ group and solvents bearing electron donor atoms (tetrahydrofuran, ethyl acetate, acetonitrile, ethanol and methanol) than the other derivatives.

**Table 4:** Equation, correlation coefficient (R^2^), slope, and intercept parameters of **TBMEBS** derivatives in eight different polarities of solvents based on solvent polarizability function ƒ(ε,n)

Method	Compound	Equation	Correlation coefficient (*R*^2^)	Slope	Intercept	Data
ῡ_a_– *f*(ε,n)	NH_2_-TBMEBS	y = -1050,9x + 32,584	0,3400	-1050,9	32,584	8
	CH_3_NH-TBMEBS	y = -736,71x + 31,409	0,5402	-736,71	31,409	8
	PhNH-TBMEBS	y = 461,27x + 32,571	0,5636	461,27	32,571	8
	o-CH_3_OPhNH-TBMEBS	y = -1186,3x + 33,253	0,1015	-1186,3	33,253	8
	p-NO_2_PhNH-TBMEBS	y = -453,3x + 27,018	0,5144	-453,3	27,018	8
	m-CF_3_PhNH-TBMEBS	y = 1336x + 33,270	0,1239	1336	33,270	8
	p-FPhNH-TBMEBS	y = -3683,4x + 33,044	0,7813	-3683,4	33,044	8
ῡ_f_– *f*(ε,n)	NH_2_-TBMEBS	y = -2928,4x + 25,656	0,8291	-2928,4	25,656	8
	CH_3_NH-TBMEBS	y = -3282,9x + 25,283	0,8489	-3282,9	25,283	8
	PhNH-TBMEBS	y = -3742,5x + 25,703	0,8860	-3742,5	25,703	8
	o-CH_3_OPhNH-TBMEBS	y = -4758,5x + 25,284	0,9334	-4758,5	25,284	8
	p-NO_2_PhNH-TBMEBS	y = -163x + 20,547	0,0062	-163	20,547	8
	m-CF_3_PhNH-TBMEBS	y = -3067,2x + 26,461	0,9052	-3067,2	26,461	8
	p-FPhNH-TBMEBS	y = -3958,2x + 25,776	0,8990	-3958,2	25,776	8
(ῡ_a_ - ῡ_f_)– *f*(ε,n)	NH_2_-TBMEBS	y = 1158,1x + 7208,2	0,3779	1158,1	7208,2	8
	CH_3_NH-TBMEBS	y = 2408,1x + 6177,6	0,6210	2408,1	6177,6	8
	PhNH-TBMEBS	y = 3647,7x + 7276,5	0,9026	3647,7	7276,5	8
	o-CH_3_OPhNH-TBMEBS	y = 4677,6x + 7819,7	0,8375	4677,6	7819,7	8
	p-NO_2_PhNH-TBMEBS	y = -177,78x + 10,418	0,0064	-177,78	10,418	8
	m-CF_3_PhNH-TBMEBS	y = 2372,6x + 9007,4	0,6384	2372,6	9007,4	8
	p-FPhNH-TBMEBS	y = 3638,3x + 6964,8	0,8939	3638,3	6964,8	8

**Fig. 5:**
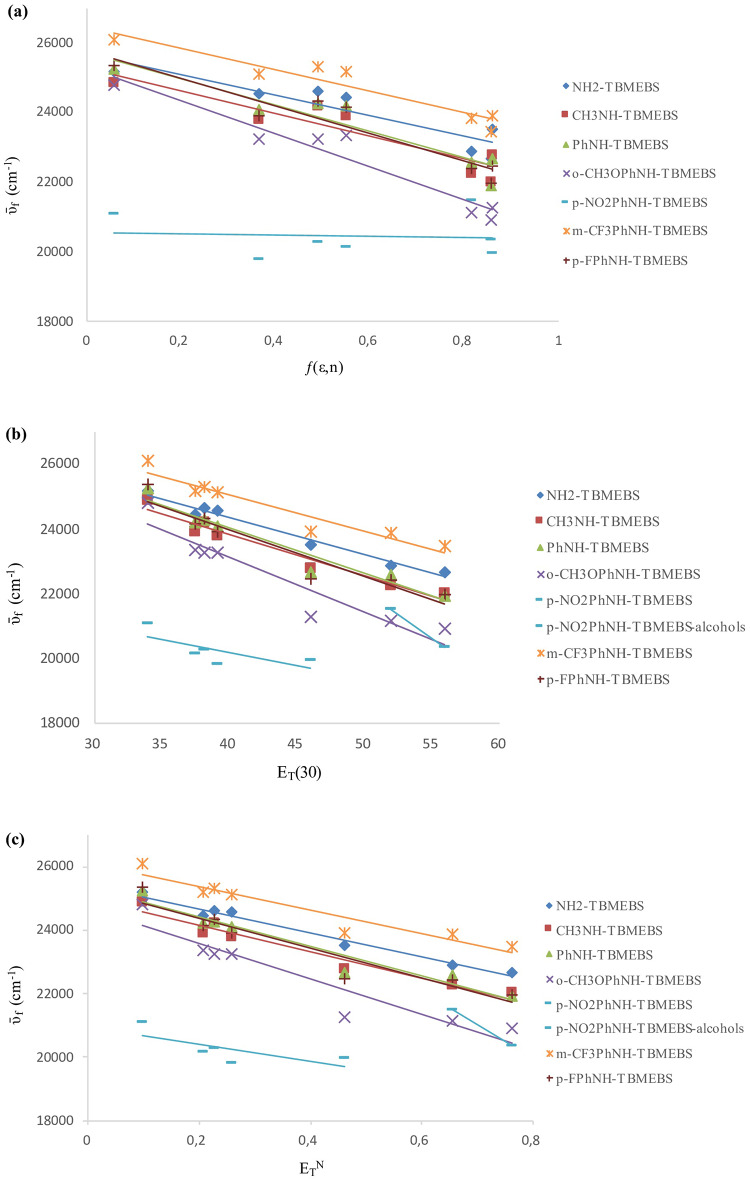
Plots of the fluorescence wavenumbers (ῡf, cm− 1) against (**a**) solvent polarizability function ƒ(ε,n) (**b**) empirical parameter values of solvent polarity ET(30) (**c**) solvent polarity parameter values ETN for TBMEBS derivatives in different solvents

**Fig. 6:**
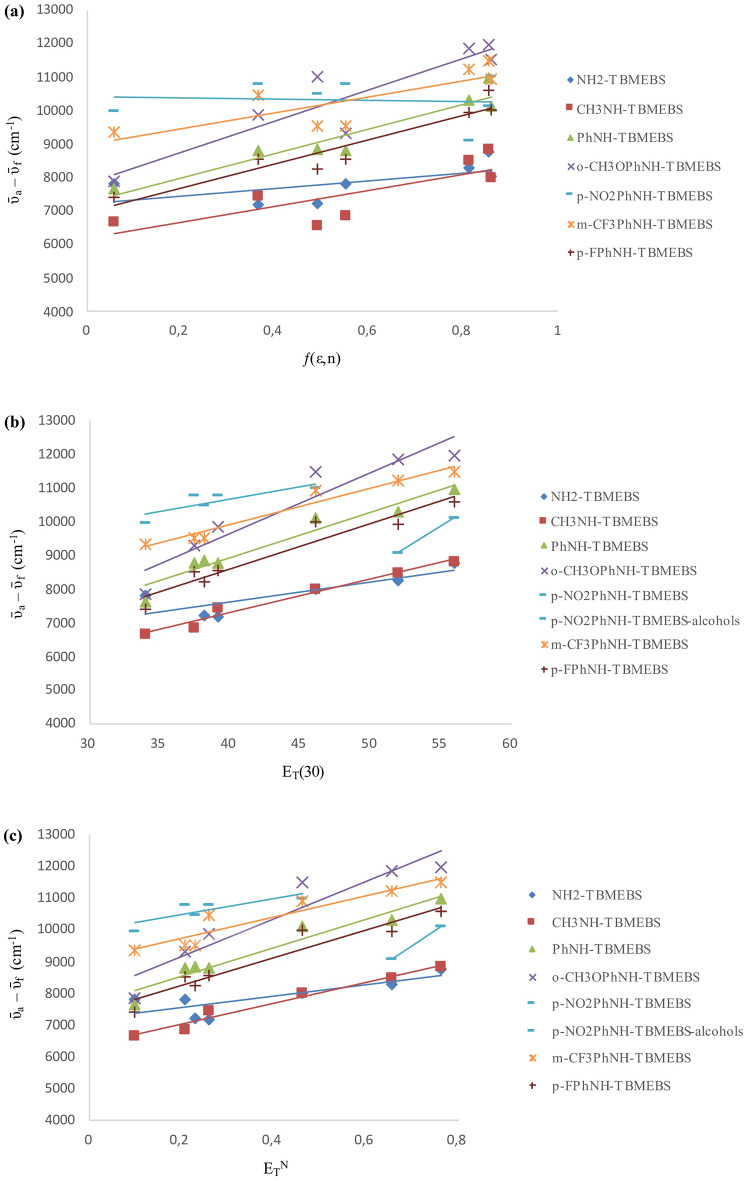
Plots of the Stokes’ shift values (Δῡ, cm− 1) against (**a**) solvent polarizability function ƒ(ε,n) (**b**) empirical parameter values of solvent polarity ET(30) (**c**) solvent polarity parameter values ETN for TBMEBS derivatives in different solvents

The solvent polarity had a greater effect on the emission than on absorption (Fig. 5). The effects of solvent polarity on the emission maxima and Stokes’ shift were investigated by plotting the fluorescence wavenumbers (ῡ_f_, cm^− 1^) (Fig. 5) and Stokes’ shifts (Δῡ = ῡ_a_ ‒ ῡ_f_, cm^− 1^) (Fig. 6) versus the ƒ(ε,n), E_T_(30), and E_T_^N^ values. The equations, correlation coefficients (R^2^), slopes, and intercepts of TBMEBS derivatives based on ƒ(ε,n), E_T_(30), and E_T_^N^ values are given in Tables 4,5, and 6, respectively. The observed lower correlations of ƒ(ε,n) demonstrated that the ƒ(ε,n) was not an adequate polarity measure for this study. The low correlation with ƒ(ε,n) may be partially attributed to the method’s lack of specific interactions between the solute and solvent [66]. The correlation is likely strengthened by molecular features such as solvation, hydrogen bond interactions, charge transfer relationships and complex formation. Both E_T_(30) and E_T_^N^ parameters can be used to evaluate the solvent polarity dependence. While E_T_(30) values provide absolute solvent polarity values, allowing the detailed analysis for specific solvent-solute interactions, E_T_^N^ values are more suitable for relative comparisons among a large scale of solvents [51]. The E_T_^N^ and E_T_(30) values were used since high E_T_^N^ and E_T_(30) values are closely associated with highly polar solvents and studied molecules containing polar groups. Thus, it was expected that there would be a more linear correlation between E_T_^N^ and E_T_(30) values of compounds and solvents. E_T_(30) and E_T_^N^ plots revealed enhanced correlations, indicating that these two parameters are better for considering the synthesized structures. The plots indicated positive solvatochromism for TBMEBS. The linear correlation is believed to be due to molecular solvation characteristics, charge transfer interactions, hydrogen bonding and complex formation [84]. Since TBMEBS have–NH groups, they can form H-bonds with solvents bearing electron donor atoms (e.g., tetrahydrofuran, ethyl acetate, acetonitrile, ethanol and methanol) and hence increase the polarizability. As the solvent polarity increased, the excited state of the TBMEBS compounds became more stable than the ground state [37]. A bathochromic shift was observed for all the TBMEBS derivatives as the solvent polarity increased. This is true with the exception of the p-NO_2_PhNH-TBMEBS compound in the polar protic solvents, which fall on a different line than the other solvents do. This is indicative of a specific solvent effect, which in this case is presumably due to hydrogen bonding of the solvent with the molecules nonbonding electrons over heteroatoms, which might affect the electron mobility toward the nitro group and hence reduce the degree of conjugation of the molecule thus yielding a blue shift in polar protic solvents in comparison to polar aprotic solvents. This deviation for p-NO_2_PhNH-TBMEBS in the polar protic solvents ethanol and methanol could also be attributed to a decrease in dipole-dipole interactions in these solvents [84]. Owing to intramolecular charge transfer, the excited state is less polar; therefore, the molecule is more stable in protic solvents than in dipolar apolar solvents. Moreover, protic solvents have greater E_T_(30) values than do the corresponding dipolar aprotic solvents [50]. In general, the E_T_^N^ and E_T_(30) values indicate the effects of polarization and H-bonding. As the solvent polarity increased, the slopes became more linear. At that rate, H-bonding and dipole-dipole interactions might occur. The positive slopes indicated that hydrogen bonding and dipole-dipole interactions became more pronounced as the solvent polarity increased. A better correlation was found when only alcohols as polar protic solvents were considered; hence, the existence of hydrogen bonding between the molecule and solvent might be considered in the presence of alcohol for TBMEBS [84]. Positive solvatochromism (a bathochromic shift) was observed as a result of the hydrogen bonding which diminished the energy of the excited state [85]. When the energy difference between the ground and excited states decreased, a bathochromic shift was observed due to hydrogen bond formation, which made the solute molecules flat and strengthened the coupling between the chromophore π-orbitals. If hydrogen bond formation disturbed the coupling between the chromophore π-orbitals, the molecule will be less conjugated and the blue shift in the spectra should have occurred. There are two types of interactions between the solute and the solvent that are responsible for the solvatochromism of the molecules. The first interaction was a nonspecific interaction between the solvent continuum and the molecules of TBMEBS, which had a major contribution to nonpolar solvents, or polar solvents, which were weak hydrogen bond acceptors. The second one originated from the formation of hydrogen bonds between the NH group of synthesized derivatives and solvent molecules, which caused a greater bathochromic shift in stronger hydrogen bond acceptors of methanol and ethanol than the other solvents. The increase in the Stokes’ shift with increasing solvent polarity indicates a greater dipole moment value for TBMEBS in the excited state than in the ground state (Fig. 6).

**Table 5:** Equation, correlation coefficient (R^2^), slope, and intercept parameters of **TBMEBS** derivatives in eight different polarities of solvents based on empirical parameter values of solvent Polarity E_T_(30)

Method	Compound	Equation	Correlation coefficient (*R*^2^)	Slope	Intercept	Data
ῡ_a_– E_T_(30)	NH_2_-TBMEBS	y = -51,752x + 34,282	0,5060	-51,752	34,282	8
	CH_3_NH-TBMEBS	y = -25,676x + 32,116	0,4922	-25,676	32,116	8
	PhNH-TBMEBS	y = 6,1601x + 32,599	0,0899	6,1601	32,599	8
	o-CH_3_OPhNH-TBMEBS	y = -55,548x + 35,100	0,1684	-55,548	35,100	8
	p-NO_2_PhNH-TBMEBS	y = -34,532x + 28,354	0,4062	-34,532	28,354	8
	m-CF_3_PhNH-TBMEBS	y = 26,249x + 33,054	0,0437	26,249	33,054	8
	p-FPhNH-TBMEBS	y = -119,25x + 36,131	0,6418	-119,25	36,131	8
ῡ_f_– E_T_(30)	NH_2_-TBMEBS	y = -115,44x + 28,975	0,9773	-115,44	28,975	8
	CH_3_NH-TBMEBS	y = -126,5x + 28,877	0,9562	-126,5	28,877	8
	PhNH-TBMEBS	y = -140,08x + 29,623	0,9416	-140,08	29,623	8
	o-CH_3_OPhNH-TBMEBS	y = -168,68x + 29,860	0,8897	-168,68	29,860	8
	p-NO_2_PhNH-TBMEBS	y = -80,13x + 23,378	0,4977	-80,13	23,378	6
	p-NO_2_PhNH-TBMEBS	y = -284,69x + 36,281	1,0000	-284,69	36,281	2
	m-CF_3_PhNH-TBMEBS	y = -112,39x + 29,568	0,9220	-112,39	29,568	8
	p-FPhNH-TBMEBS	y = -144,32x + 29,756	0,9066	-144,32	29,756	8
(ῡ_a_ - ῡ_f_)– E_T_(30)	NH_2_-TBMEBS	y = 59,607x + 5245,6	0,6882	59,607	5245,6	8
	CH_3_NH-TBMEBS	y = 99,919x + 3313,9	0,9738	99,919	3313,9	8
	PhNH-TBMEBS	y = 135,08x + 3519,3	0,9389	135,08	3519,3	8
	o-CH_3_OPhNH-TBMEBS	y = 179,87x + 2477,1	0,8890	179,87	2477,1	8
	p-NO_2_PhNH-TBMEBS	y = 74,972x + 7687,8	0,6719	74,972	7687,8	6
	p-NO_2_PhNH-TBMEBS	y = 261,39x − 4490,3	1,0000	261,39	-4490,3	2
	m-CF_3_PhNH-TBMEBS	y = 107,97x + 5604,1	0,9577	107,97	5604,1	8
	p-FPhNH-TBMEBS	y = 133,77x + 3258,7	0,9167	133,77	3258,7	8

**Table 6:** Equation, correlation coefficient (R^2^), slope, and intercept parameters of **TBMEBS** derivatives in eight different polarities of solvents based on solvent Polarity parameter values E_T_^N^

Method	Compound	Equation	Correlation coefficient (*R*^2^)	Slope	Intercept	Data
ῡ_a_– E_T_^N^	NH_2_-TBMEBS	y = -1290,1x + 32,478	0,3559	-1290,1	32,478	8
	CH_3_NH-TBMEBS	y = -726,4x + 31,266	0,3647	-726,4	31,266	8
	PhNH-TBMEBS	y = 411,88x + 32,677	0,3120	411,88	32,677	8
	o-CH_3_OPhNH-TBMEBS	y = -929,15x + 32,932	0,0432	-929,15	32,932	8
	p-NO_2_PhNH-TBMEBS	y = -421,81x + 26,920	0,3093	-421,81	26,920	8
	m-CF_3_PhNH-TBMEBS	y = 2004,3x + 33,266	0,1936	2004,3	33,266	8
	p-FPhNH-TBMEBS	y = -3716,5x + 32,364	0,5523	-3716,5	32,364	8
ῡ_f_– E_T_^N^	NH_2_-TBMEBS	y = -3817,2x + 25,445	0,9782	-3817,2	25,445	8
	CH_3_NH-TBMEBS	y = -4181x + 25,008	0,9562	-4181	25,008	8
	PhNH-TBMEBS	y = -4618,6x + 25,334	0,9371	-4618,6	25,334	8
	o-CH_3_OPhNH-TBMEBS	y = -5568,6x + 24,698	0,8876	-5568,6	24,698	8
	p-NO_2_PhNH-TBMEBS	y = -2738,1x + 20,947	0,5141	-2738,1	20,947	6
	p-NO_2_PhNH-TBMEBS	y = -10643x + 28,466	1,0000	-10,643	28,466	2
	m-CF_3_PhNH-TBMEBS	y = -3706,8x + 26,128	0,9181	-3706,8	26,128	8
	p-FPhNH-TBMEBS	y = -4760,9x + 25,338	0,9031	-4760,9	25,338	8
(ῡ_a_ - ῡ_f_)– E_T_^N^	NH_2_-TBMEBS	y = 1805,6x + 7179,2	0,6379	1805,6	7179,2	8
	CH_3_NH-TBMEBS	y = 3300,4x + 6371,5	0,9733	3300,4	6371,5	8
	PhNH-TBMEBS	y = 4455,8x + 7654,4	0,9352	4455,8	7654,4	8
	o-CH_3_OPhNH-TBMEBS	y = 5941x + 7981,3	0,8885	5941	7981,3	8
	p-NO_2_PhNH-TBMEBS	y = 2544x + 9966,7	0,6845	2544	9966,7	6
	p-NO_2_PhNH-TBMEBS	y = 9771,4x + 2685,2	1,0000	9771,4	2685,2	2
	m-CF_3_PhNH-TBMEBS	y = 3355,5x + 9078,9	0,8867	3355,5	9078,9	8
	p-FPhNH-TBMEBS	y = 4407,9x + 7355,6	0,9111	4407,9	7355,6	8

**Fig. 7:**
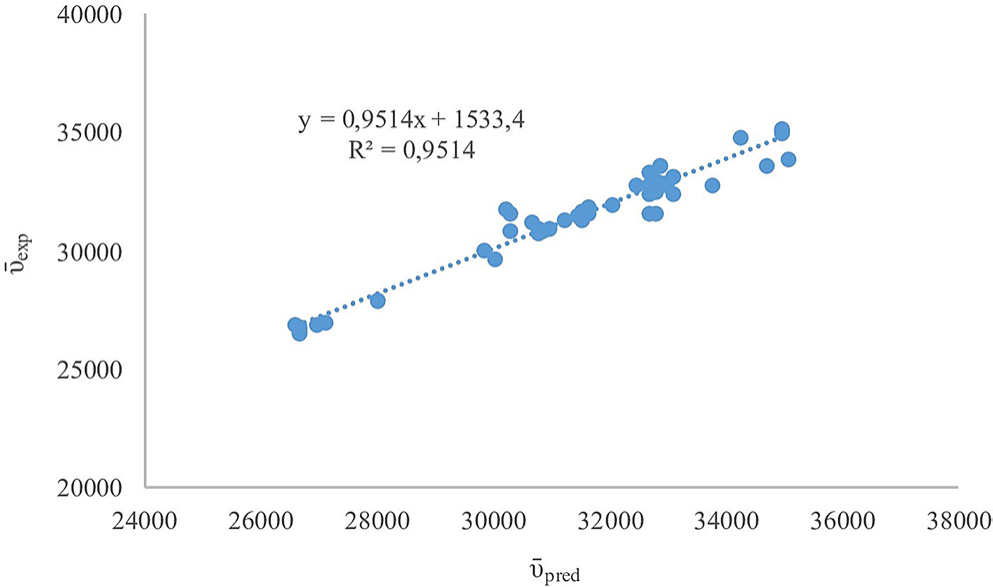
Plot of the experimental absorption maxima wavenumbers (ῡexp) and predicted absorption maxima wavenumbers (ῡpred) of TBMEBS derivatives based on Kamlet-Taft solvent parameters

The Kamlet-Taft parameters describe solvent effects based on solvatochromic behaviour [86]. The correlation between the experimental absorption maxima wavenumbers (ῡ_exp_) and the predicted ones (ῡ_pred_) is demonstrated in Fig. 7 and resulted in an R^2^ value of 0,9514 indicating that the Kamlet-Taft model represented the solute-solvent interactions, particularly H-bonding and polarizability effects successfully. Generally, methanol and ethanol as polar protic solvents and tetrahydrofuran and acetonitrile as strong H-bond acceptors affected the spectral shifts significantly through H-bonding. NH_2_-TBMEBS and CH_3_-TBMEBS containing the H-bond donor -NH groups showed decreased ῡ_abs_ values in protic solvents, ethanol and methanol with high β values (hydrogen-bond acceptor) through H-bonding [87]. The ability of these molecules to form H-bonds with protic solvents makes stable solute-solvent complexes and leads to a red shift of the absorption band maximum [88]. PhNH-TBMEBS exhibited minor shifts with modest solvent sensitivity. The compound contains an amine group with lone electron pairs attached to the benzene ring which participated in resonance with π-system of the benzene [89]. o-CH_3_OPhNH-TBMEBS showed large ῡ_abs_ values particularly in polar solvents with strong dipole-dipole interactions. The electron-donating methoxy substituent in the molecule increased electron density and solvent polarity response [90]. p-NO_2_PhNH-TBMEBS and m-CF_3_PhNH-TBMEBS bearing nitro and trifluoromethyl substituents in the benzene ring as a strong electron-withdrawing group enhanced intramolecular polarization while diminishing the intermolecular H-bonding, causing relatively consistent ῡ_abs_ values, particularly in the case of p-NO_2_PhNH-TBMEBS [91–94]. p-NO_2_PhNH-TBMEBS exhibited a limited change in ῡ_abs_ values with solvent polarity which indicated that intramolecular charge transfer (ICT) dominated the solvent effects by strong internal polarization leading to the low solvent response [95]. The m-CF_3_PhNH-TBMEBS compound displayed significant changes in ῡ_abs_ in different solvents with relatively high ῡ_abs_ in nonpolar solvents and sharp decreases in polar solvents. This behaviour was attributed to the excited π-state which was highly sensitive to solvent polarity and susceptibility to the -CF_3_ group’s moderate electron-withdrawing nature [96]. The p-FPhNH-TBMEBS compound substituted with fluorine as a weak electron-withdrawing group enhancing the π–π* interactions, caused high ῡ_abs_ values in π-rich solvents like toluene and tetrahydrofuran and significant red-shifts in protic solvents such as ethanol and methanol indicating moderate H-bonding sensitivity [97].

**Fig. 8:**
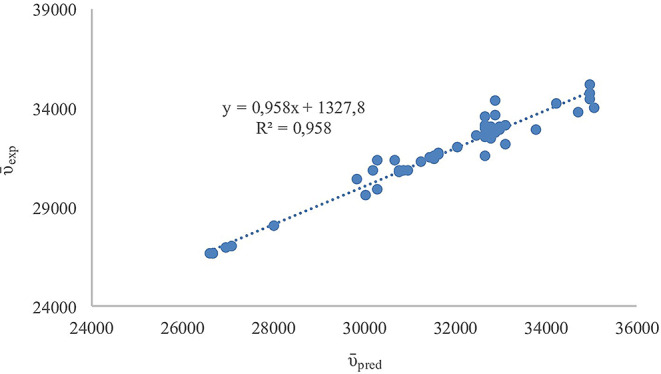
Plot of the experimental absorption maxima wavenumbers (ῡexp) and predicted absorption maxima wavenumbers (ῡpred) of TBMEBS derivatives based on Catalán solvent parameters

The Catalán solvent polarity model was employed to provide a better understanding of solute and solvent interactions of the studied compounds through their spectroscopic behaviour. A strong correlation between the experimental absorption maxima wavenumbers (ῡ_exp_) and those calculated from the model (ῡ_pred_) produced a correlation coefficient (R^2^) 0,958 as shown in Fig. 8. Both NH_2_-TBMEBS and CH_3_NH-TBMEBS which contain aliphatic amino groups (–NH_2_ and–NHCH_3_, respectively) exhibited notable negative responses to solvent basicity (SB) and dipolarity (SdP). The replacement of the hydrogen atom in the amino group (–NH_2_) with a methyl group (–NHCH_3_) reduced the H-bonding ability of CH_3_NH-TBMEBS and increased sensitivity to solvent polarizability (SP) while NH_2_-TBMEBS showed strong hydrogen bond donating ability [98–100]. According to the Catalán analysis, both PhNH-TBMEBS and p-FPhNH-TBMEBS displayed a negative effect from solvent polarizability (SP), particularly stronger for PhNH-TBMEBS. PhNH-TBMEBS promotes a planar and delocalized system, favouring interactions more via polarizability and π‒π interaction [101]. The presence of electron-withdrawing fluorine in FPhNH-TBMEBS enhanced the molecular planarity and electron delocalization, increasing solvent polarizability (SP) sensitivity and solvent basicity (SB) responsiveness [102]. Although both p-NO_2_PhNH-TBMEBS and m-CF_3_PhNH-TBMEBS feature strong electron-withdrawing groups of–NO_2_ and–CF_3_, respectively, their Catalán responses differ significantly. The para substituted–NO_2_ derivative increased molecular polarity, resulting in strong negative sensitivity to solvent polarizability (SP) and dipolarity, aligning well with the Catalán model [103]. In contrast, the meta substituted–CF_3_ derivative showed a poor model with highly positive solvent acidity (SA) and basicity (SB) due to both steric hindrance and electronic effects that weakened solute-solvent interaction [104]. The ortho-OCH_3_ substituted o-CH_3_OPhNH-TBMEBS showed a strong negative response to solvent dipolarity (SdP), positive responses to solvent acidity (SA), basicity (SB), and polarizability (SP) with a moderate Catalán model fit. These features indicated that the ortho methoxy group might induced intramolecular hydrogen bonding and steric hindrance, resulting in shielding the polar groups from solvent interactions [105, 106]. These findings emphasize that the position and nature of substituent on the aromatic ring play a significant role in determining solute-solvent interactions.

The investigation of dipole moment for compounds bearing various electron-donating and electron-withdrawing substituents is essential for the design of fluorophores, as the donor-acceptor system and conjugation effect their photophysical properties and solvatochromic behaviour [107]. Due to the presence of electron-donating amino group at the position-4 and electron-withdrawing benzenesulfonamide group at the position-4 of the thiazole ring, NH_2_-TBMEBS is expected to have a moderately high dipole moment, arising from effective intramolecular charge transfer and good conjugation from the donor (-NH_2_) toward the acceptor benzenesulfonamide [107]. Both CH_3_NH-TBMEBS and PhNH-TBMEBS promote donor-acceptor structures featuring electron-donating methylamino and phenylamino groups at the position-2, respectively, and electron-withdrawing benzenesulfonamide group at the position-4 of the thiazole ring are expected to exhibit moderately strong dipole moment. Due to the inductive effect of the methyl group, reducing electron-donating ability of the ‒NHCH_3_ group, it can show lower dipole moment than NH_2_-TBMEBS [108]. The dipole moment of PhNH-TBMEBS is predicted to be lower than that one of NH_2_-TBMEBS, as the conjugation may be reduced by the non-planarity of the bulky phenyl ring [109]. Although o-CH_3_OPhNH-TBMEBS contains the electron-doating methoxyphenylamino substituent at the position-2 of the thiazole ring, the ortho position causes steric hinderance, leading to non-planarity, thereby diminishing donor-acceptor conjugation and resulting in a moderate dipole moment [110]. p-NO_2_PhNH-TBMEBS featuring the strong electron-withdrawing nitro group on the para-substituted phenyl ring at the position-2 of the thiazole ring is expected to exhibit a high dipole moment as the nitro group enhances intramolecular charge transfer in conjunction with the sulfonamide moiety, amplifying the molecular polarity [111]. m-CF_3_PhNH-TBMEBS is predicted to have a moderate to high dipole moment due to the electron-withdrawing trifluoromethyl group and its meta positioning, reducing electron density on the phenyl ring with limited conjugation with the thiazole core [112]. p-FPhNH-TBMEBS is anticipated to introduce a moderate dipole moment due to a balance between the electron-donating resonance of the amino group and the electron-withdrawing inductive effect of the para-substituted fluorine atom [113]. In summary, the dipole moment estimation of seven thiazole-based derivatives demonstrates how substituent electronic characteristic and molecular geometry at the thiazole ring influence charge transfer and conjugation, providing insights for designing fluorophores with solvent polarity sensitivity.

**Fig. 9:**
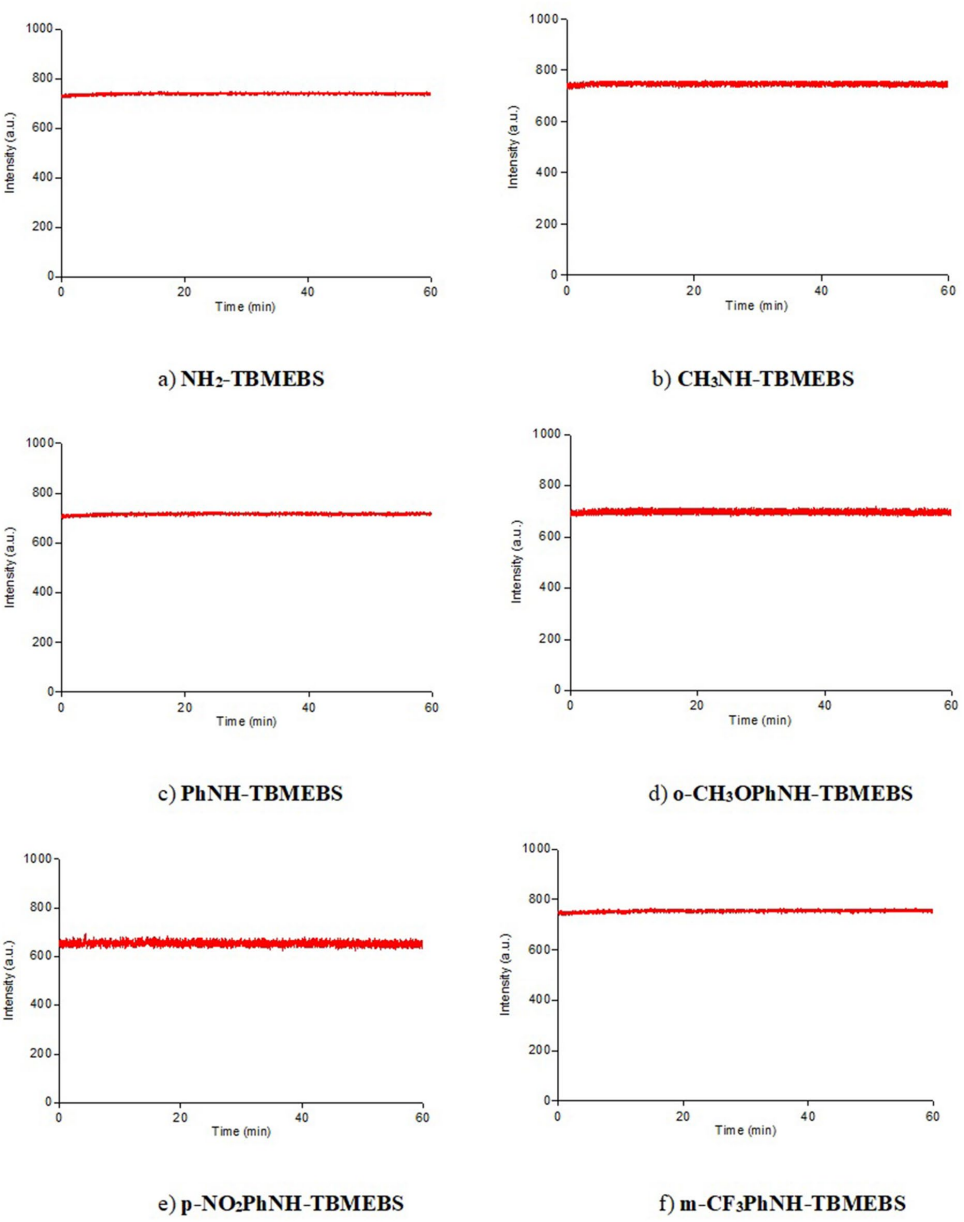
The photostability test results of **a**) NH2-TBMEBS, **b**) CH3NH-TBMEBS, **c**) PhNH-TBMEBS, **d**) o-CH3OPhNH-TBMEBS, **e**) p-NO2PhNH-TBMEBS, and f) m-CF3PhNH-TBMEBS in ethanol with the concentration of 1 × 10− 5 M

Photostability is one of the characteristics of fluorophores that determines their fluorescence properties. The photostability test results of NH_2_-TBMEBS, CH_3_NH-TBMEBS, PhNH-TBMEBS, o-CH_3_OPhNH-TBMEBS, p-NO_2_PhNH-TBME, and m-CF_3_PhNH-TBMEBS in ethanol with the concentration of 1 × 10^− 5^ M are given in Fig. 9 as examples. The photostability test results were recorded for the TBMEBS compounds in eight solvents after 1 h of monitoring under continuous excitation with intense light from a high power Xe lamb. All the TBMEBS derivatives exhibited excellent photostabilities in all the solvents tested.

## Conclusions

A series of novel thiazole derivatives containing a benzenesulfonamide moiety and a bis(2-methoxyethyl) group with electron-donating substituents (NH_2_-TBMEBS, CH_3_NH-TBMEBS, PhNH-TBMEBS, o-CH_3_OPhNH-TBMEBS) and electron-withdrawing substituents (p-NO_2_PhNH-TBMEBS, m-CF_3_PhNH-TBMEBS, p-FPhNH-TBMEBS) were synthesized via the Hantzsch thiazole synthesis method, purified, and confirmed by structure. The fluorescence characteristics of the novel synthesized thiazole molecules containing different kinds of electron donor and acceptor groups in the benzene ring were investigated to understand how the structure of the molecules affects their properties and hence provide more information about the potential uses of these substances in fluorescence sensing technologies. The synthesized thiazole derivatives exhibited moderate Stokes’ shift values and quantum yield values and excellent photostabilities. In general, a large molar extinction coefficient and Stokes’ shift were accompanied by high quantum yield values. While NH_2_-TBMEBS and CH_3_NH-TBMEBS presented one maximum absorption wavelength value, PhNH-TBMEBS, o-CH_3_OPhNH-TBMEBS, p-NO_2_PhNH-TBMEBS, m-CF_3_PhNH-TBMEBS, and p-FPhNH-TBMEBS presented two maximum absorption wavelength values. All compounds presented maximum emission wavelengths in the range of 362–542 nm, and their Stokes’ shift values ranged between 5020 and 11,974 cm⁻¹. The first absorption maxima were assumed to be related to the π–π^*^ transition of the benzene group conjugation, and the second absorption maxima belonged to the n–π^*^ transition of the imine group (–C = N–) conjugation in the thiazole nucleus. The solvatochromic behaviors of all the TBMEBS derivatives were investigated according to the solvent polarizability ƒ(ε,n), empirical parameters of solvent polarity E_T_(30), and solvent polarity E_T_^N^ parameters. Linear correlations revealed that there might be interactions of H-bonding and dipole-dipole forces in mostly polar electron donating and protic solvents. The Kamlet-Taft solvatochromic analysis showed that the type of substituents on benzene ring and solvent polarity played a significant role in the solvent-solute interactions. Electron-withdrawing groups such as–NO_2_ and–CF_3_ groups suppressed H-bonding formation but enhanced dipole-dipole interactions while electron-donating groups such as–OCH_3_ increased the molecule’s sensitivity to solvent polarity. The Catalán solvent polarity analysis revealed that the type, position, electronic character, and steric factor of the functional group on the studied compounds affected solute-solvent interactions significantly as captured by their sensitivity to Catalán solvent parameters (SA, SB, SdP, and SP).

The thiazole derivatives bearing a benzenesulfonamide moiety represent a unique class of heterocyclic compounds that combine the electronic versatility of the thiazole ring with the functional adaptability of the sulfonamide group, making it a valuable scaffold in medicinal chemistry and material sciences.

These conjugated systems are highly favorable for the development of fluorescent probes, sensing materials, and therapeutic agents, owing to their capacity for intramolecular charge transfer, sensitivity to solvent environments (solvatochromism), and adjustable photophysical characteristics. The extended conjugation between the thiazole core and benzenesulfonamide group plays a crucial role in fine-tuning their emission behavior, making them ideal for fluorescence-based detection and imaging platforms.

Moreover, the open chain bis(2-methoxyethyl) moieties are flexible so that they can orient themselves in space to make effective interactions with metal ions, so the synthesized derivatives can also be applied as fluoroionophores.

## Data Availability

No datasets were generated or analysed during the current study.
